# Supplementary material for: Rare β-Resorcylic Acid Derivatives from a Halophyte-Associated Fungus Colletotrichum gloeosporioides JS0419 and Their Antifungal Activities
Source: Mar Drugs. 2022 Mar 7;20(3):195. doi: 10.3390/md20030195 (PMC8951769; doi:10.3390/md20030195)
Supplement: Supplementary file 1 [file marinedrugs-20-00195-s001.zip › marinedrugs-1615334-supplementary.pdf]

Supporting Information

Rare β-resorcylic acid derivatives from a halophyte-associated fungus *Colletotrichum gloeosporioides* JS0419 and their antifungal activities

Sunghee Bang [1,†], Jaekyeong Kim [2,†], Jiwon Oh [1], Ji-Seok Kim [3], Seong-Ryong Yu [3], Stephen T. Deyrup [4], Yong-Sun Bahn [3] and Sang Hee Shim [2,*]

[1]College of Pharmacy, Duksung Women's University, Seoul 01369, Republic of Korea

[2]Natural Products Research Institute, College of Pharmacy, Seoul National University, Seoul 08826, Republic of Korea

[3]Department of Biotechnology, College of Life Science and Biotechnology, Yonsei University, Seoul 03722, Republic of Korea

[4]Department of Chemistry and Biochemistry, Siena College, Londonville, NY12211, USA

*Correspondence: sanghee_shim@snu.ac.kr; Tel.: 82-2-880-2479 (S.H.S.)

† Both authors equally contributed to this work.

Table of Contents

Figure S1. HPLC-UV chromatogram of the initial EtOAc extract.

Figure S2. ^{1}H NMR spectrum (500 MHz, CD_3OD) of (**1**)

Figure S3. ^{13}C NMR spectrum (125 MHz, CD_3OD) of (**1**)

Figure S4. ^{1}H-^{1}H COSY spectrum (500 MHz, CD_3OD) of (**1**)

Figure S5. HSQC spectrum (500 MHz, CD_3OD) of (**1**)

Figure S6. HMBC spectrum (500 MHz, CD_3OD) of (**1**)

Figure S7. ^{1}H NMR spectrum (500 MHz, CD_3OD) of acetylated derivative of (**1**)

Figure S8. HETLOC spectrum (800 MHz, CD_3OD) of (**1**)

Figure S9. ^{1}H NMR spectrum (500 MHz, CD_3OD) of acetonide product (**1a**)

Figure S10. ^{13}C NMR spectrum (125 MHz, CD_3OD) of acetonide product (**1a**)

Figure S11. HMBC spectrum (500 MHz, CD_3OD) of acetonide product (**1a**)

Figure S12. ^{1}H NMR spectrum (500 MHz, CD_3OD) of *S*-MTPA ester (**1b**)

Figure S13. ^{1}H-^{1}H COSY spectrum (500 MHz, CD_3OD) of *S*-MTPA ester (**1b**)

Figure S14. ^{1}H NMR spectrum (500 MHz, CD_3OD) of *R*-MTPA ester (**1c**)

Figure S15. ^{1}H-^{1}H COSY spectrum (500 MHz, CD_3OD) of *R*-MTPA ester (**1c**)

Figure S16. HRESIMS spectrum of (**1**)

Figure S17. ^{1}H NMR spectrum (500 MHz, CD_3OD) of (**2**)

Figure S18. ^{13}C NMR spectrum (125 MHz, CD_3OD) of (**2**)

Figure S19. ^{1}H-^{1}H COSY spectrum (500 MHz, CD_3OD) of (**2**)

Figure S20. HSQC spectrum (500 MHz, CD_3OD) of (**2**)

Figure S21. HMBC spectrum (500 MHz, CD_3OD) of (**2**)

Figure S22. HRESIMS spectrum of (**2**)

Figure S23. ^{1}H NMR spectrum (500 MHz, CD_3OD) of (**3**)

Figure S24. ^{13}C NMR spectrum (125 MHz, CD_3OD) of (**3**)

Figure S25. ^{1}H-^{1}H COSY spectrum (500 MHz, CD_3OD) of (**3**)

Figure S26. HSQC spectrum (500 MHz, CD_3OD) of (**3**)

Figure S27. HMBC spectrum (500 MHz, CD_3OD) of (**3**)

Figure S28. HETLOC spectrum (800 MHz, CD_3OD) of (**3**)

Figure S29. ^{1}H NMR spectrum (500 MHz, CD_3OD) of acetonide product (**3a**)

Figure S30. ^{13}C NMR spectrum (125 MHz, CD_3OD) of acetonide product (**3a**)

Figure S31. HMBC spectrum (500 MHz, CD_3OD) of acetonide product (**3a**)

Figure S32. HRESIMS spectrum of (**3**)

Figure S33. ^{1}H NMR spectrum (500 MHz, CD_3OD) of (**4**)

Figure S34. ^{13}C NMR spectrum (125 MHz, CD_3OD) of (**4**)

Figure S35. ^{1}H-^{1}H COSY spectrum (500 MHz, CD_3OD) of (**4**)

Figure S36. HSQC spectrum (500 MHz, CD_3OD) of (**4**)

Figure S37. HMBC spectrum (500 MHz, CD_3OD) of (**4**)

Figure S38. HRESIMS spectrum of (**4**)

Figure S39. ^{1}H NMR spectrum (500 MHz, CD_3OD) of (**5**)

Figure S40. ^{13}C NMR spectrum (125 MHz, CD_3OD) of (**5**)

Figure S41. ^{1}H-^{1}H COSY spectrum (500 MHz, CD_3OD) of (**5**)

Figure S42. HSQC spectrum (500 MHz, CD_3OD) of (**5**)

Figure S43. HMBC spectrum (500 MHz, CD_3OD) of (**5**)

Figure S44. HRESIMS spectrum of (**5**)

Figure S45. ^{1}H NMR spectrum (500 MHz, CD_3OD) of (**6**)

Figure S46. ^{13}C NMR spectrum (125 MHz, CD_3OD) of (**6**)

Figure S47. ^{1}H-^{1}H COSY spectrum (500 MHz, CD_3OD) of (**6**)

Figure S48. HSQC spectrum (500 MHz, CD_3OD) of (**6**)

Figure S49. HMBC spectrum (500 MHz, CD_3OD) of (**6**)

Figure S50. HRESIMS spectrum of (**6**)

Figure S51. ^{1}H NMR spectrum (500 MHz, CD_3OD) of (**8**)

Figure S52. ^{13}C NMR spectrum (125 MHz, CD_3OD) of (**8**)

Figure S53. ^{1}H-^{1}H COSY spectrum (500 MHz, CD_3OD) of (**8**)

Figure S54. HSQC spectrum (500 MHz, CD_3OD) of (**8**)

Figure S55. HMBC spectrum (500 MHz, CD_3OD) of (**8**)

Figure S56. HRESIMS spectrum of (**8**)

Figure S1. HPLC-UV chromatogram of the initial EtOAc extract.

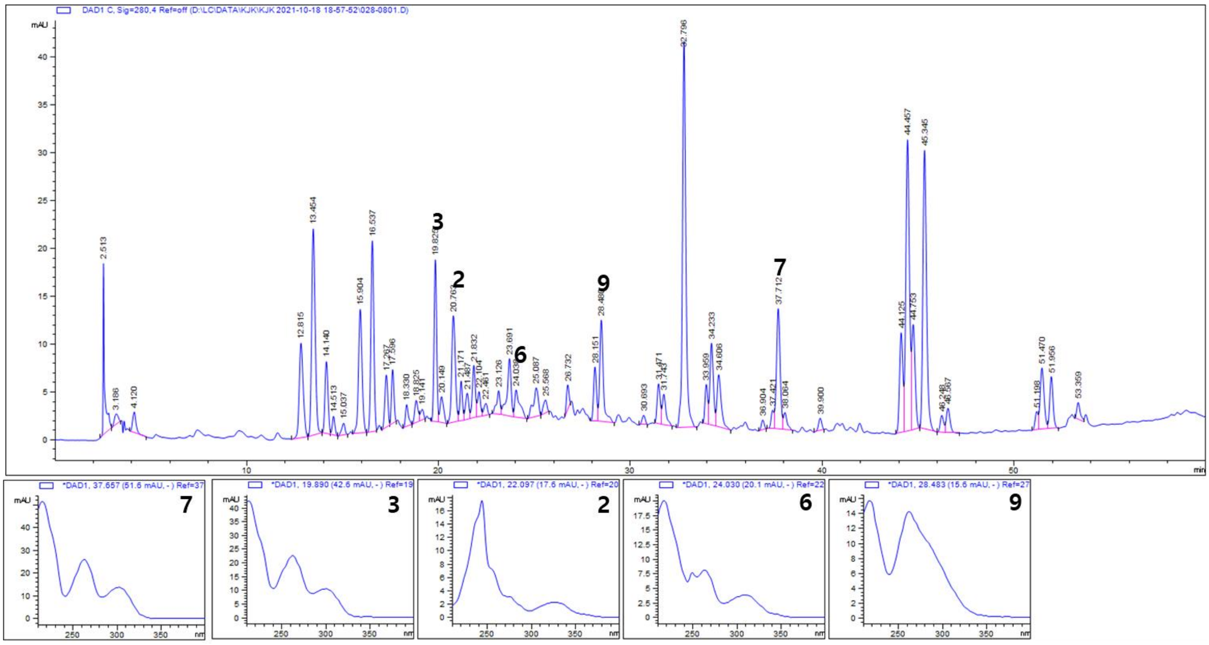

Figure S2. ^{1}H NMR spectrum (500 MHz, CD_3OD) of (**1**)

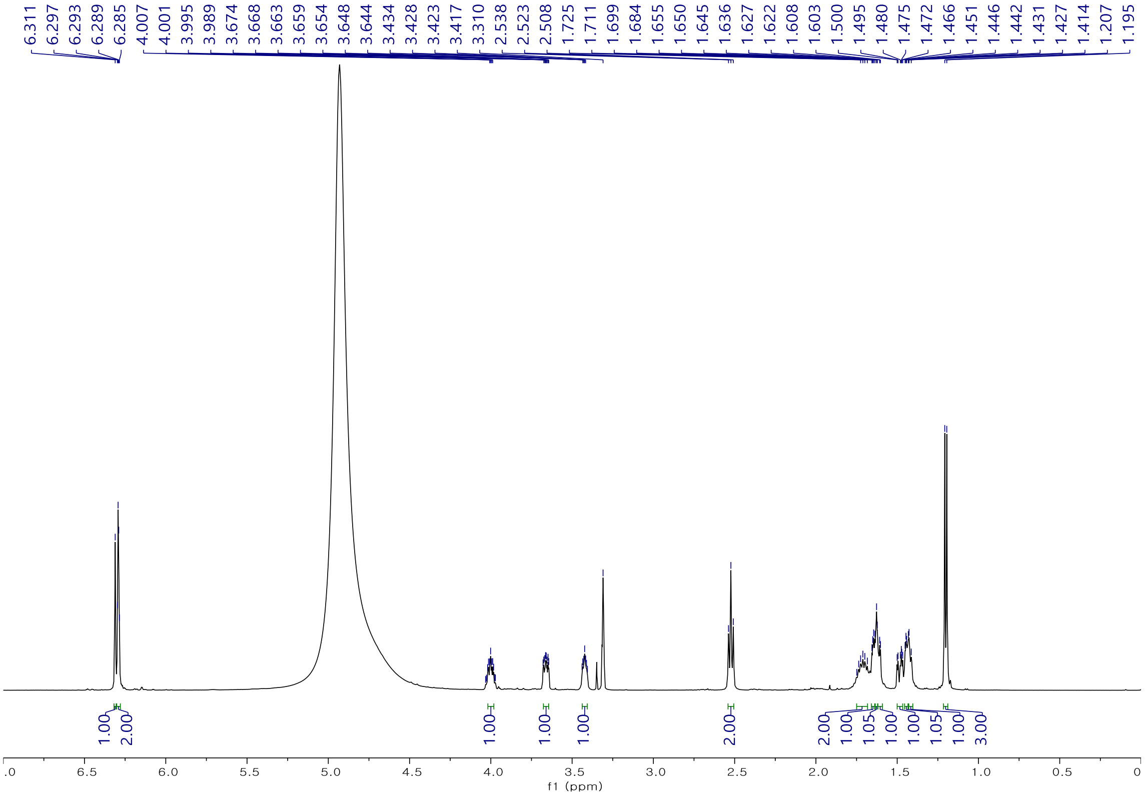

Figure S3. ^{13}C NMR spectrum (125 MHz, CD_3OD) of (**1**)

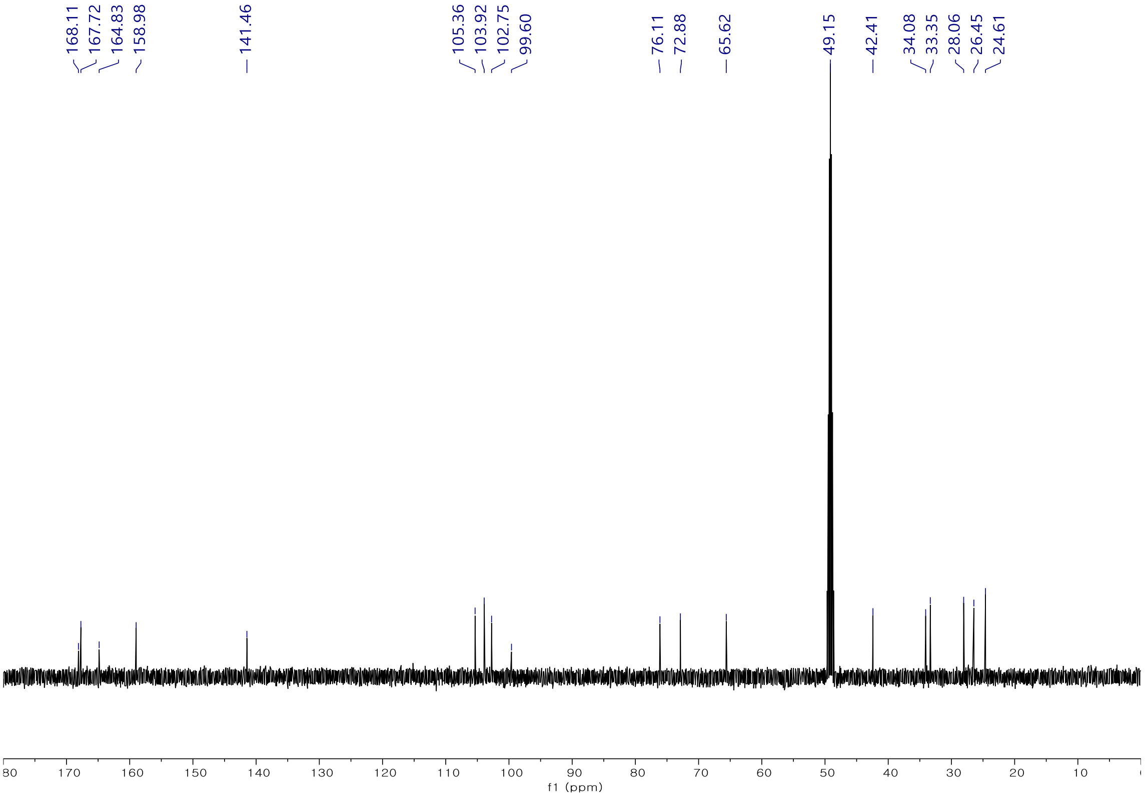

Figure S4. ^{1}H-^{1}H COSY spectrum (500 MHz, CD_3OD) of (**1**)

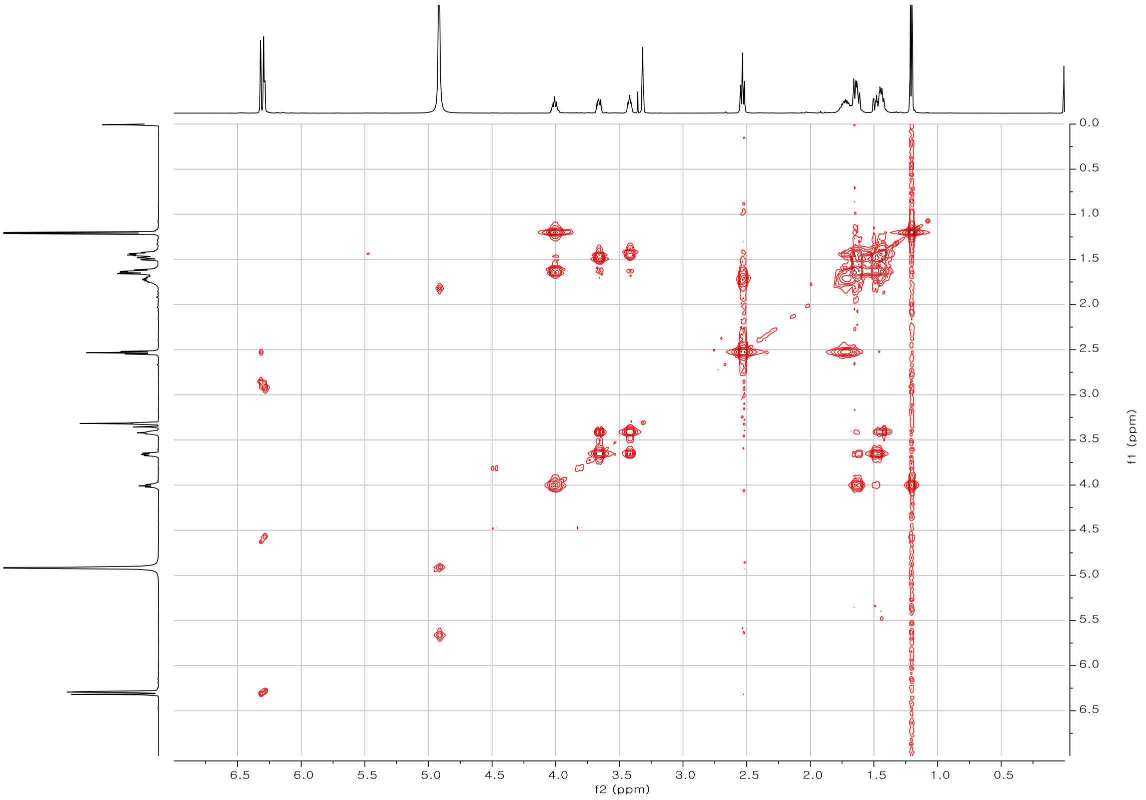

Figure S5. HSQC spectrum (500 MHz, CD_3OD) of (**1**)

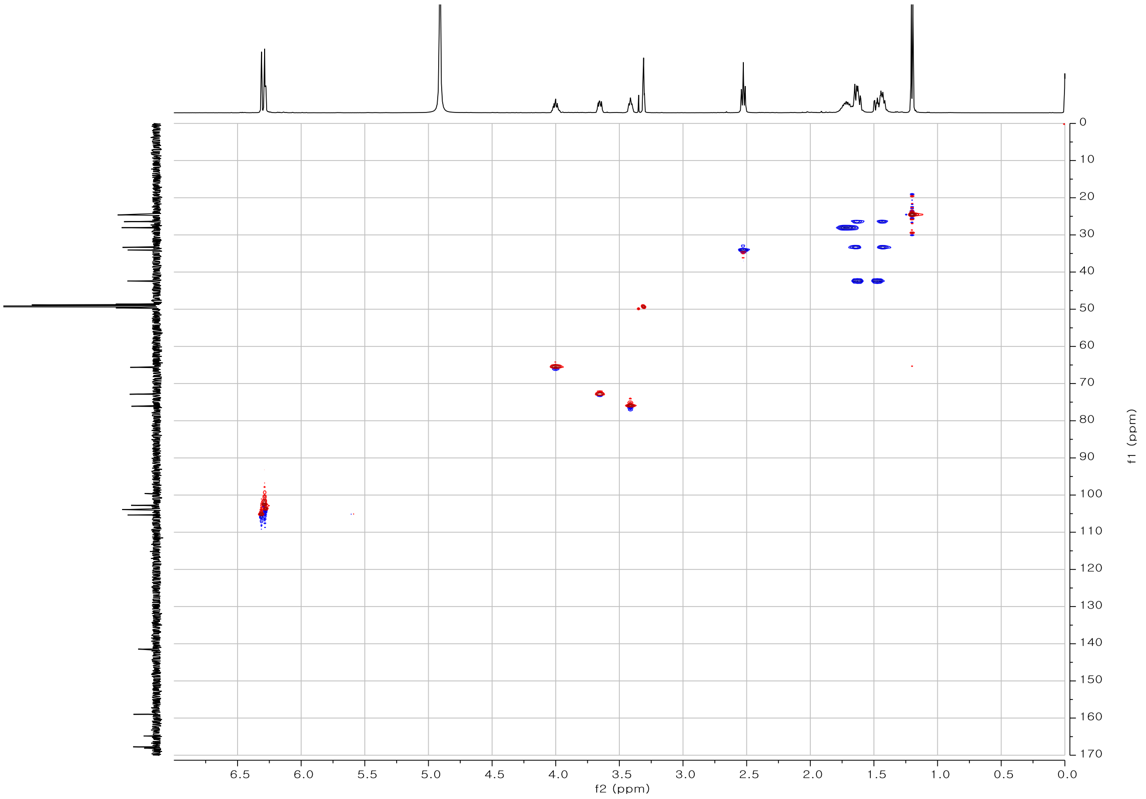

Figure S6. HMBC spectrum (500 MHz, CD3OD) of (**1**)

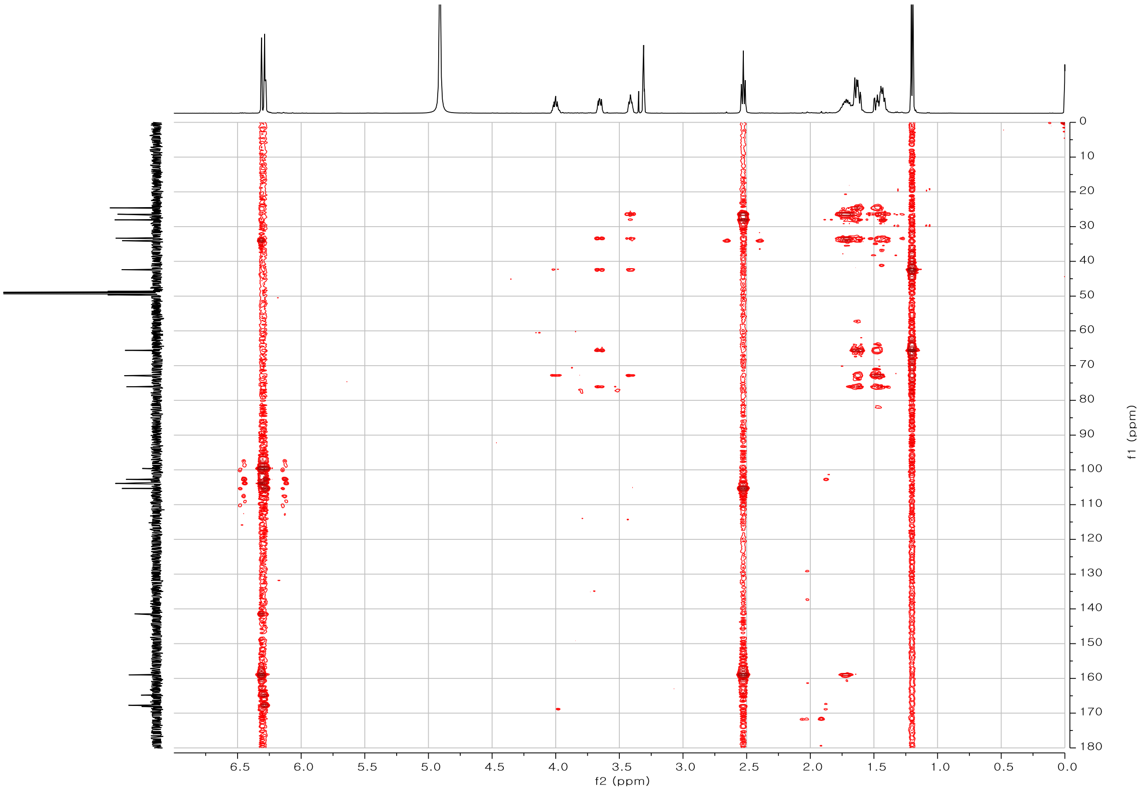

Figure S7. ^{1}H NMR spectrum (500 MHz, CD3OD) of acetylated derivative of (**1**)

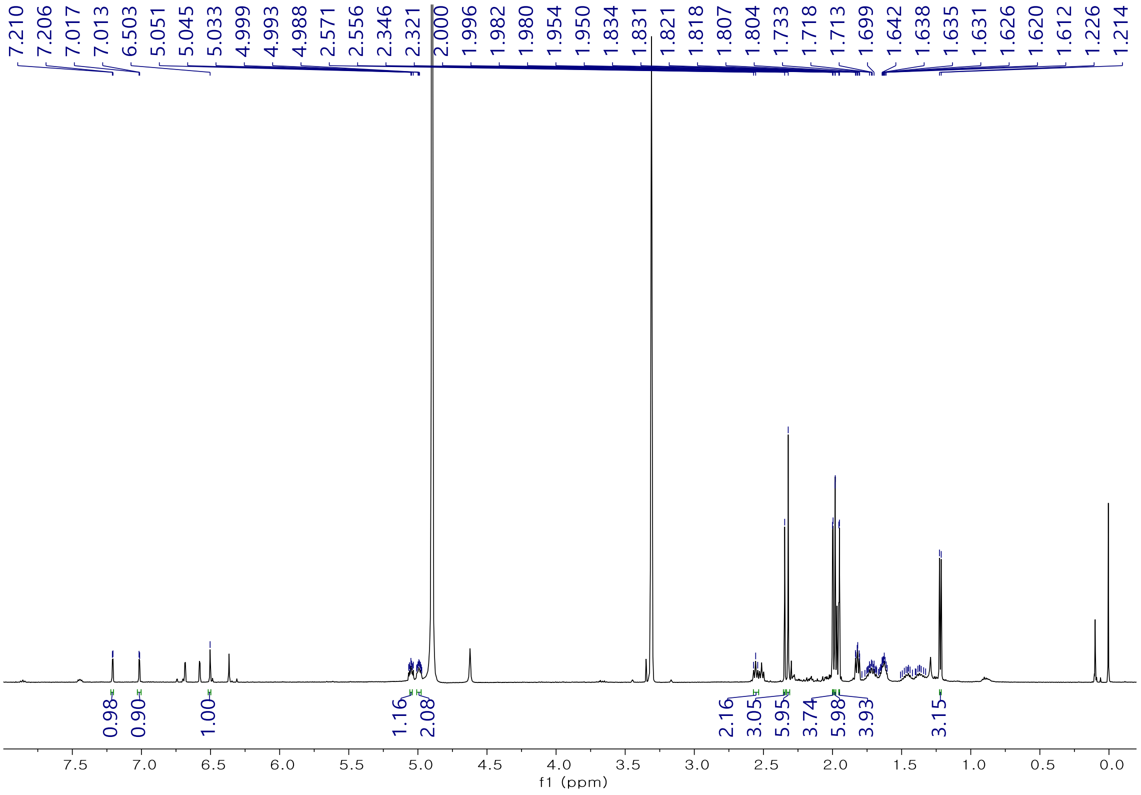

Figure S8. HETLOC spectrum (800 MHz, CD_3OD) of (**1**)

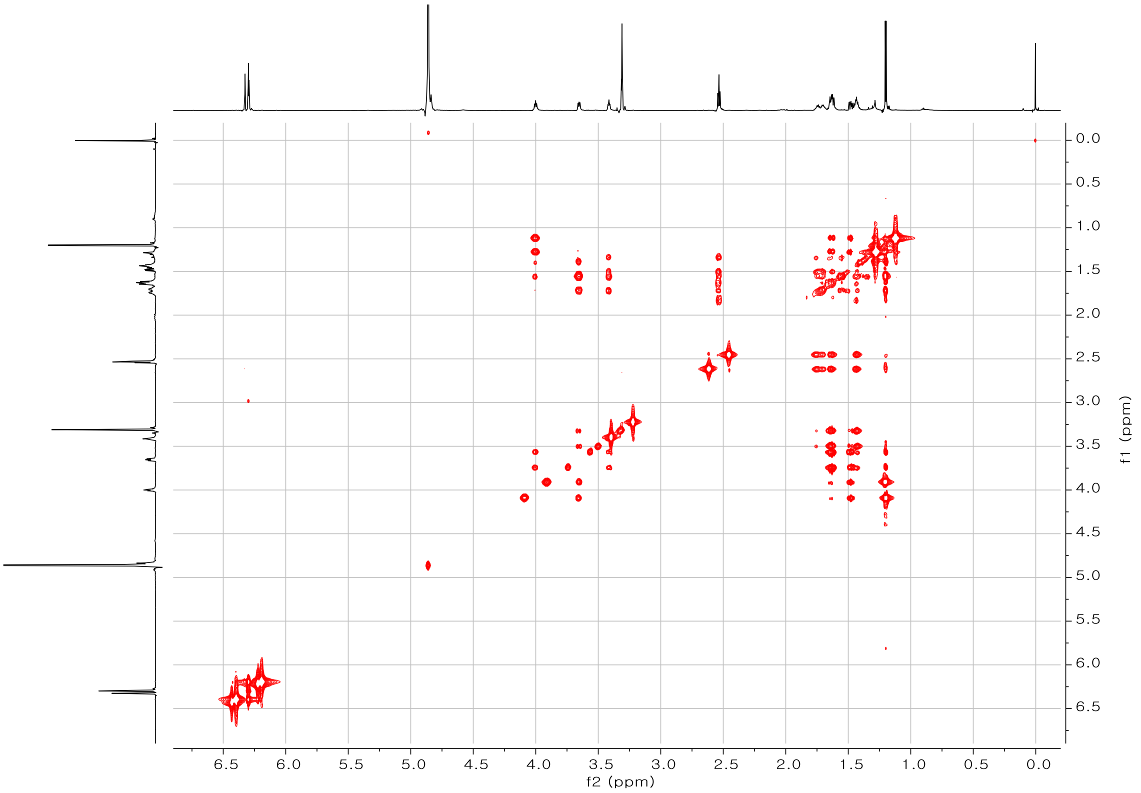

Figure S9. ^{1}H NMR spectrum (500 MHz, CD_3OD) of acetonide product (**1a**)

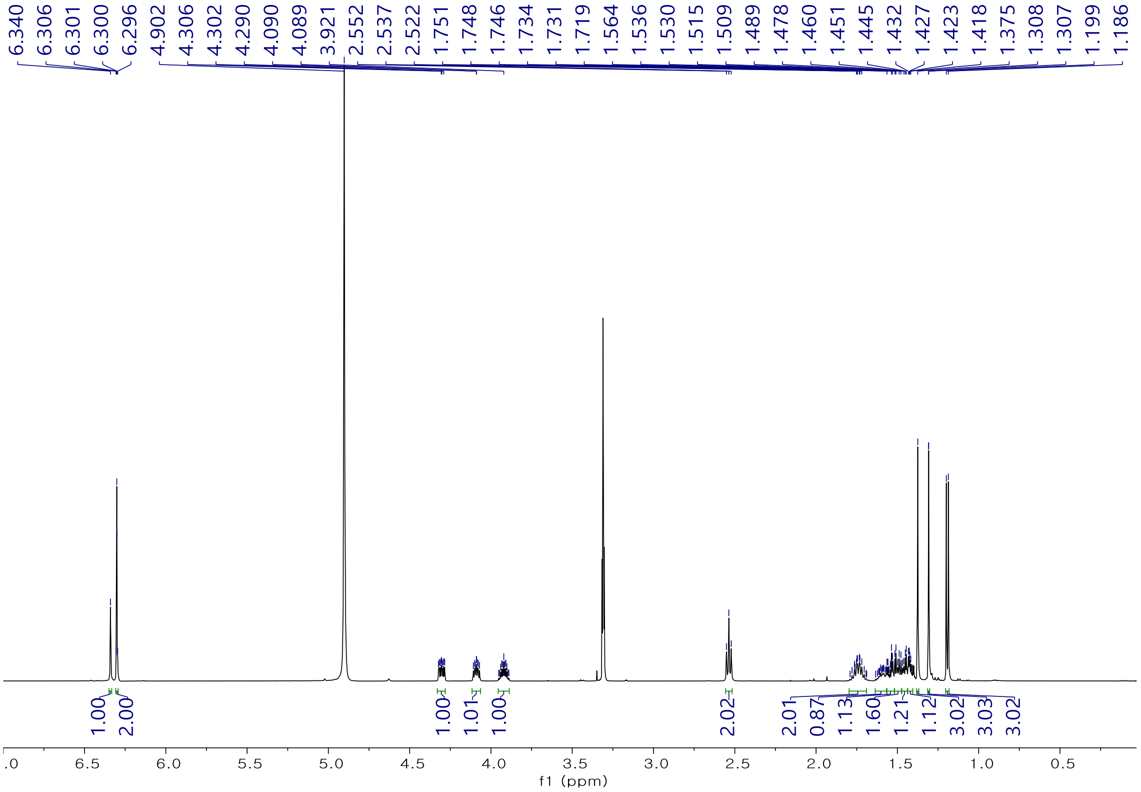

Figure S10. ^{13}C NMR spectrum (125 MHz, CD_3OD) of acetonide product (**1a**)

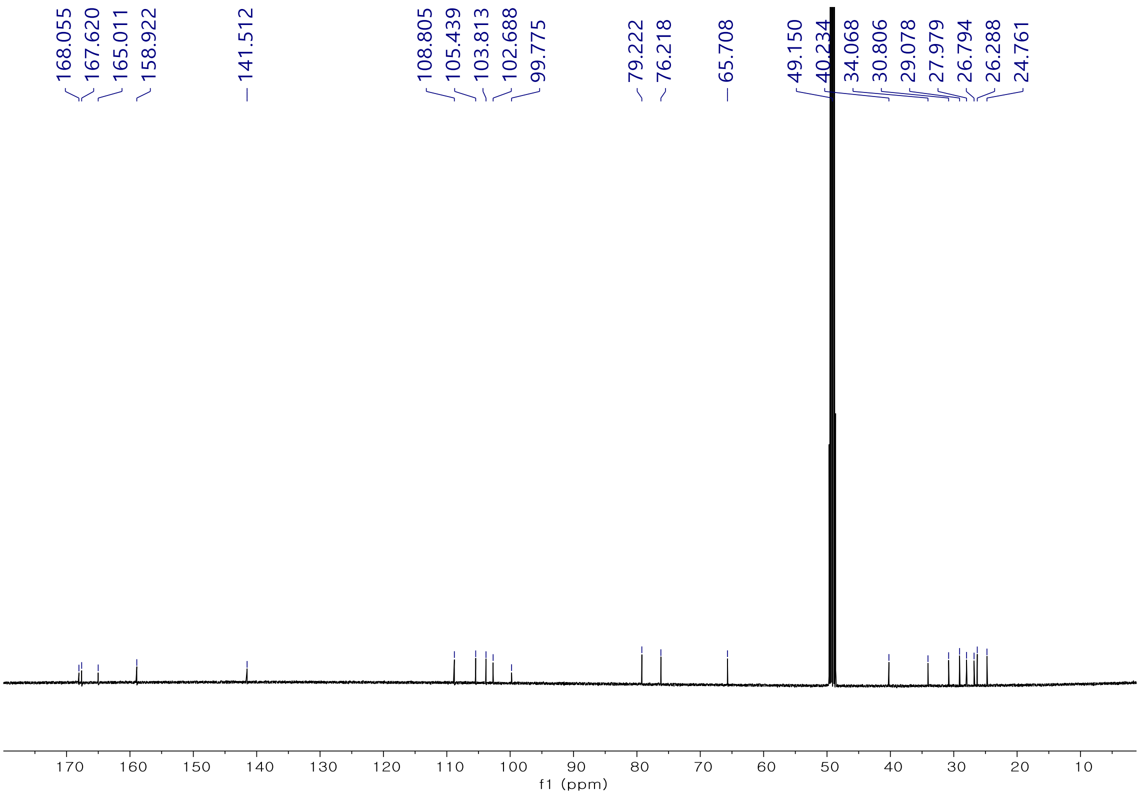

Figure S11. HMBC spectrum (500 MHz, CD_3OD) of acetonide product (**1a**)

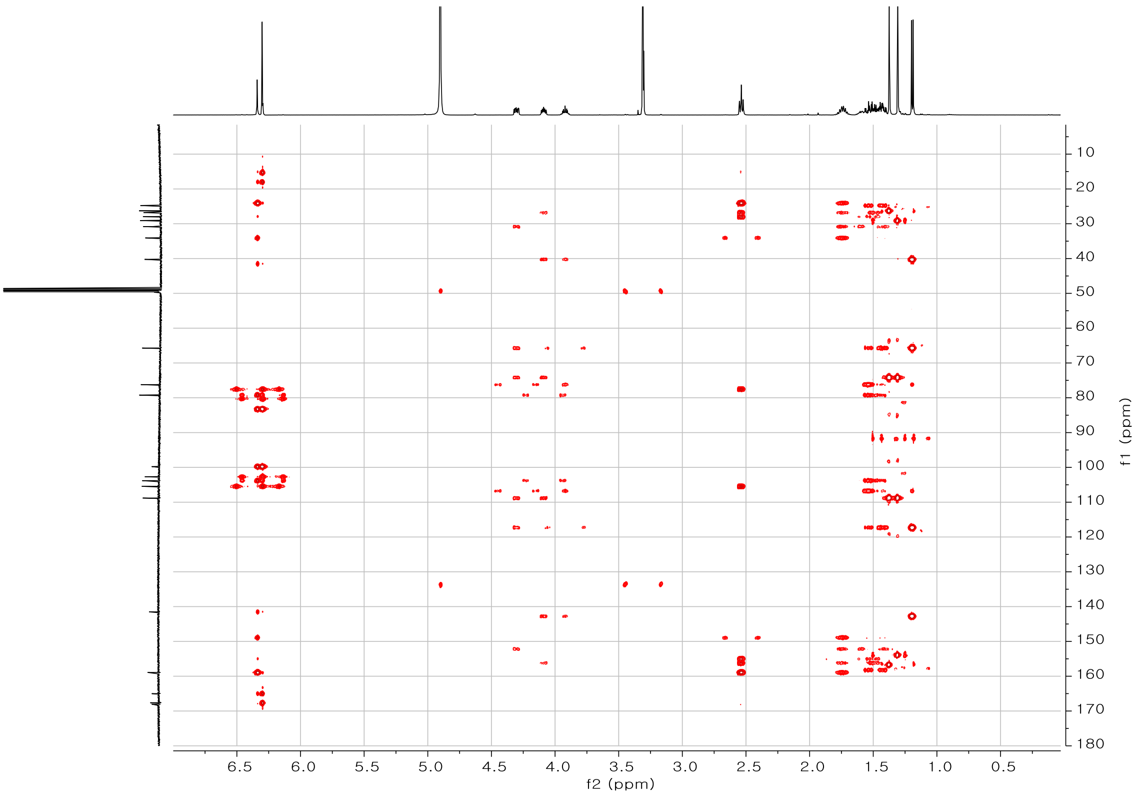

Figure S12. ^{1}H NMR spectrum (500 MHz, CD_3OD) of *S*-MTPA ester (**1b**)

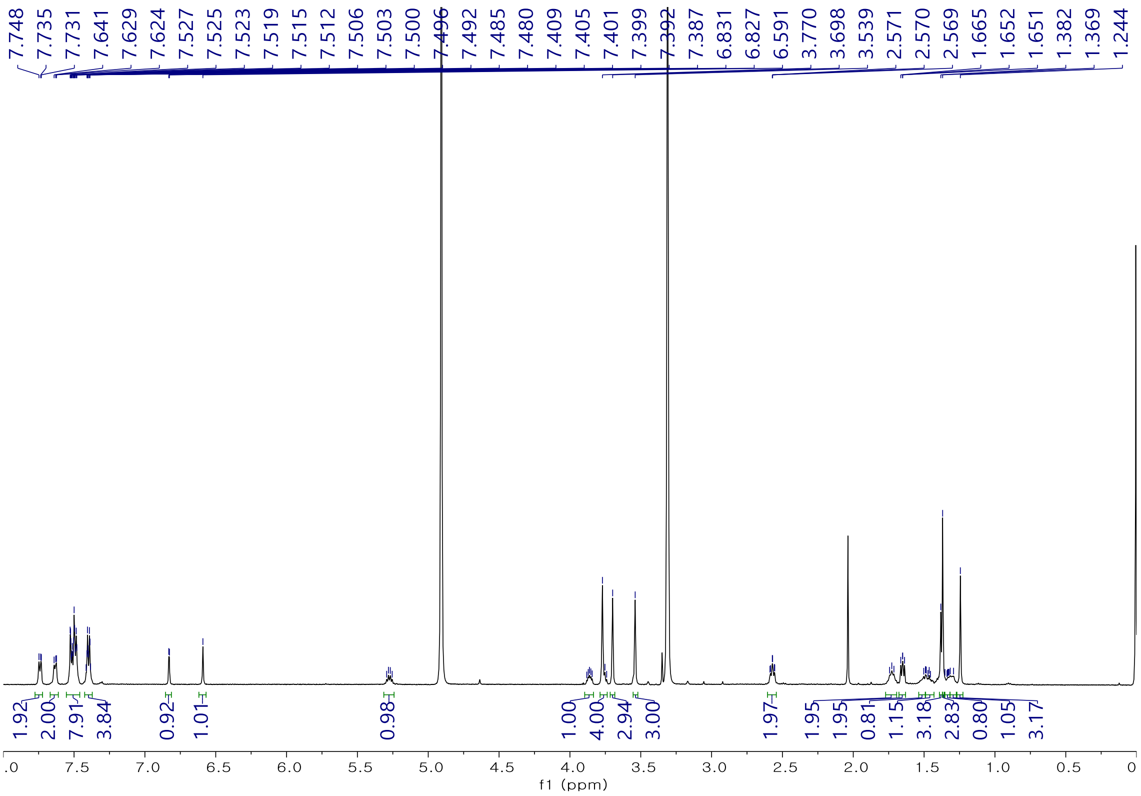

Figure S13. ^{1}H-^{1}H COSY spectrum (500 MHz, CD_3OD) of *S*-MTPA ester (**1b**)

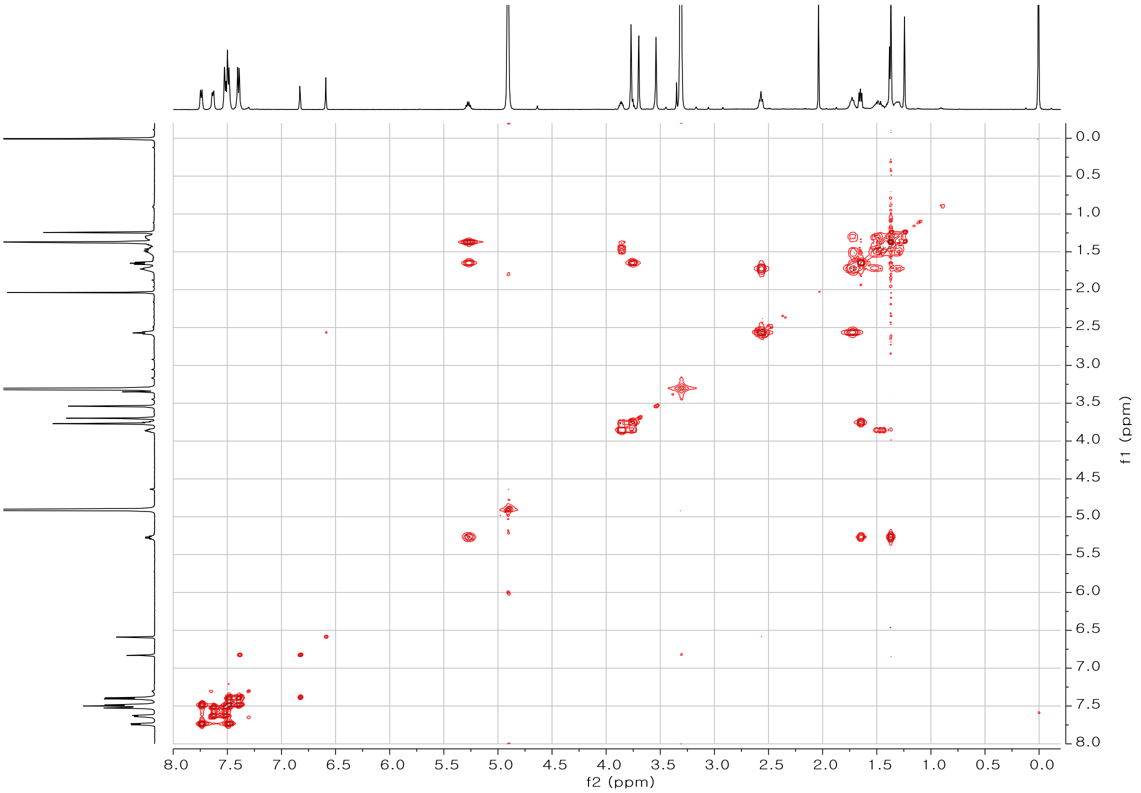

Figure S14. ^{1}H NMR spectrum (500 MHz, CD_3OD) of *R*-MTPA ester (**1c**)

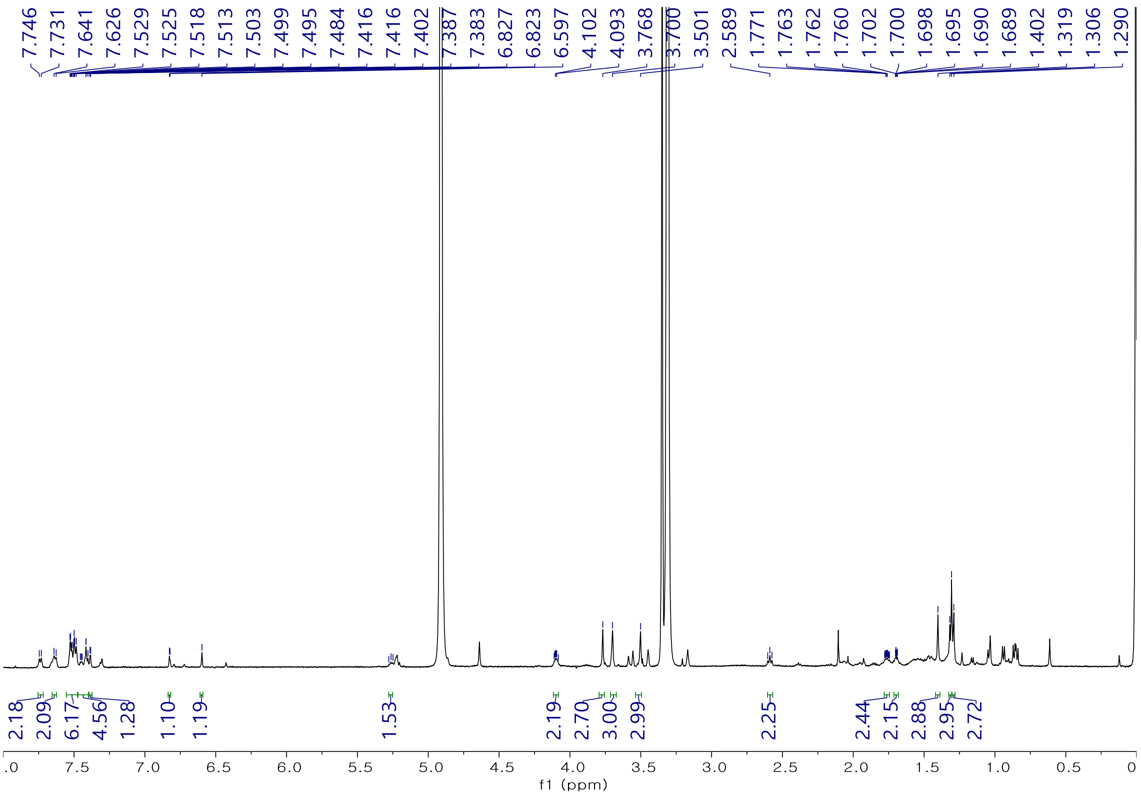

Figure S15. ^{1}H-^{1}H COSY spectrum (500 MHz, CD_3OD) of *R*-MTPA ester (**1c**)

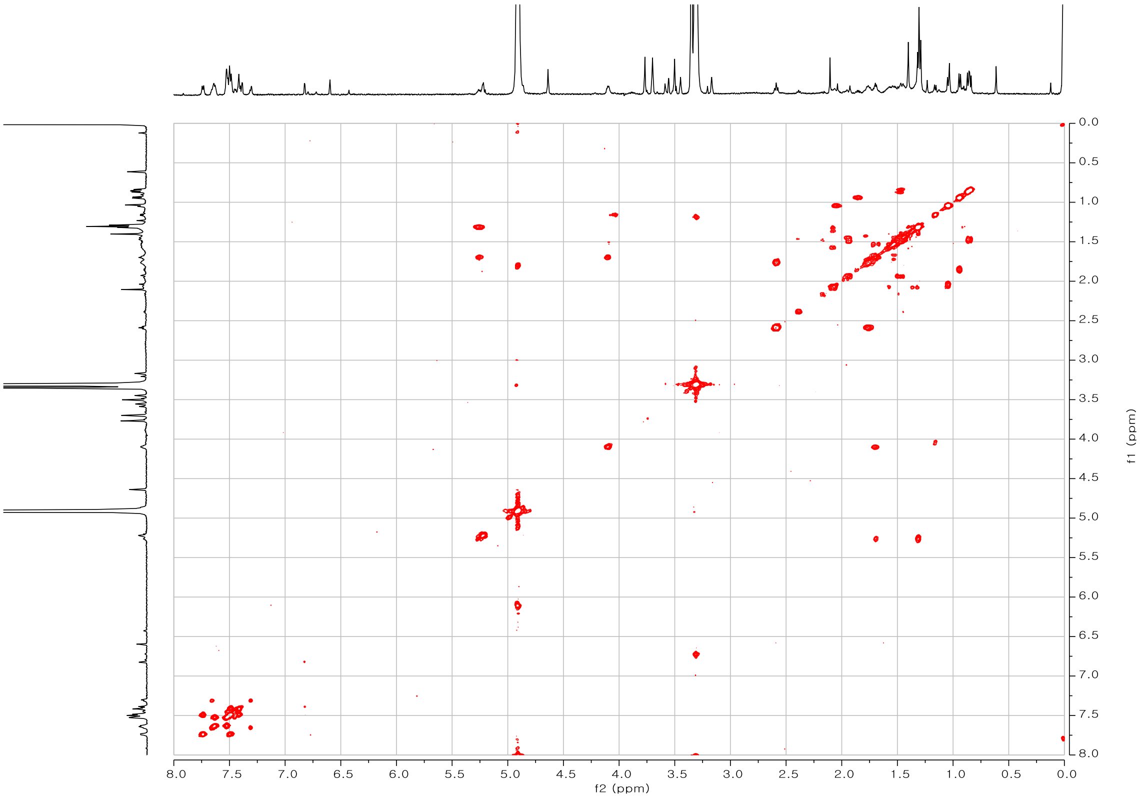

Figure S16. HRESIMS spectrum of (**1**)

Compound Spectrum SmartFormula Report

Analysis Info

Analysis Name	D:\Data\Data\2019\DSWU\SSH\BSH06\BSH001.d	Acquisition Date	6/27/2019 11:36:58 AM
Method	Tune_pos_Standard_100_600_190102.d.m	Operator	lee
Sample Name	BSH01_9F2E	Instrument	compact 8255754.10024
Comment			

Acquisition Parameter

Source Type	ESI	Ion Polarity	Positive	Set Nebulizer	0.4 Bar
Focus	Active	Set Capillary	4000 V	Set Dry Heater	200 °C
Scan Begin	100 m/z	Set End Plate Offset	-500 V	Set Dry Gas	4.0 l/min
Scan End	600 m/z	Set Charging Voltage	2000 V	Set Divert Valve	Source
		Set Corona	0 nA	Set APCI Heater	0 °C

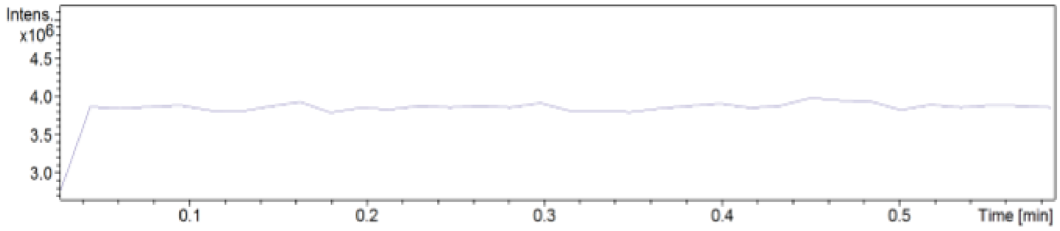

#	RT [min]	Area	Int. Type	I	S/N	Chromatogram	Max. m/z	FWHM [min]
n.a.	0.4	n.a.	Average spectrum	n.a.	n.a.	n.a.	375.1420	n.a.

+MS, 0.4min #21

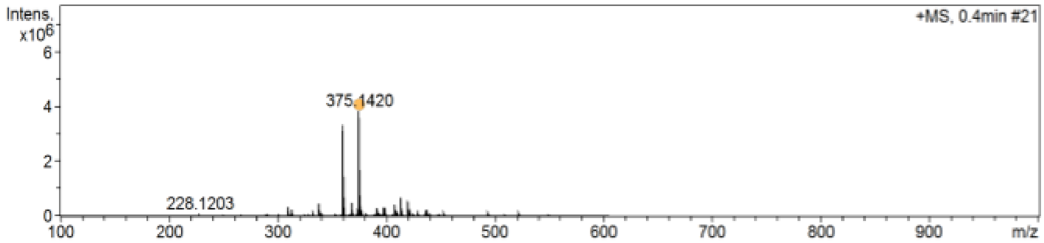

Meas. m/z	#	Ion Formula	m/z	err [ppm]	mSigma	# Sigma	Score	rdb	e⁻ Conf	N-Rule
375.1420	1	C20H23O7	375.1438	-4.8	4.9	1	61.52	9.5	even	ok
	2	C17H15N10O	375.1425	1.2	6.5	2	100.00	15.5	even	ok
	3	C16H19N6O5	375.1411	2.4	8.5	3	77.03	10.5	even	ok
	4	C21H19N4O3	375.1452	8.4	15.9	4	15.89	14.5	even	ok
	5	C15H23N2O9	375.1398	5.9	18.9	5	33.88	5.5	even	ok
	1	C19H20N4NaO3	375.1428	1.9	6.0	1	71.44	11.5	even	ok
	2	C18H24NaO7	375.1414	1.6	6.7	2	100.00	6.5	even	ok
	3	C15H16N10NaO	375.1401	-5.2	9.7	3	30.66	12.5	even	ok
	4	C14H20N6NaO5	375.1387	8.8	21.2	4	7.39	7.5	even	ok

Figure S17. ^{1}H NMR spectrum (500 MHz, CD_3OD) of (**2**)

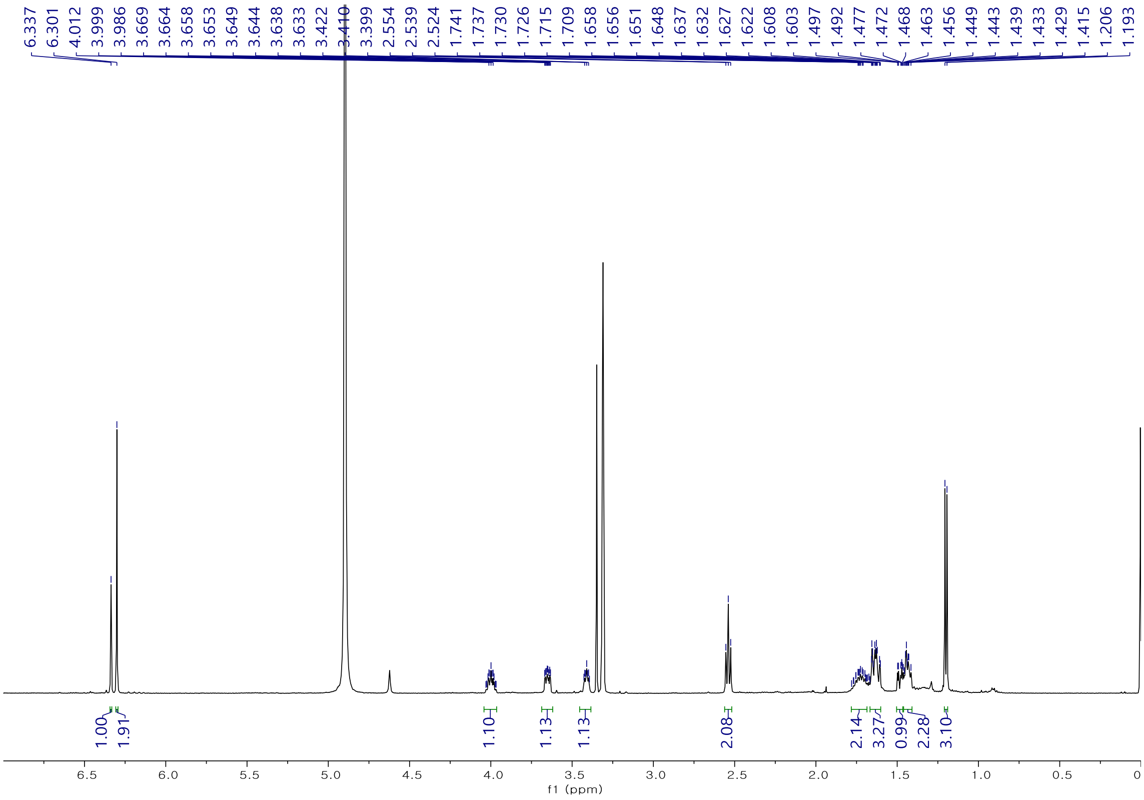

Figure S18. ^{13}C NMR spectrum (125 MHz, CD_3OD) of (**2**)

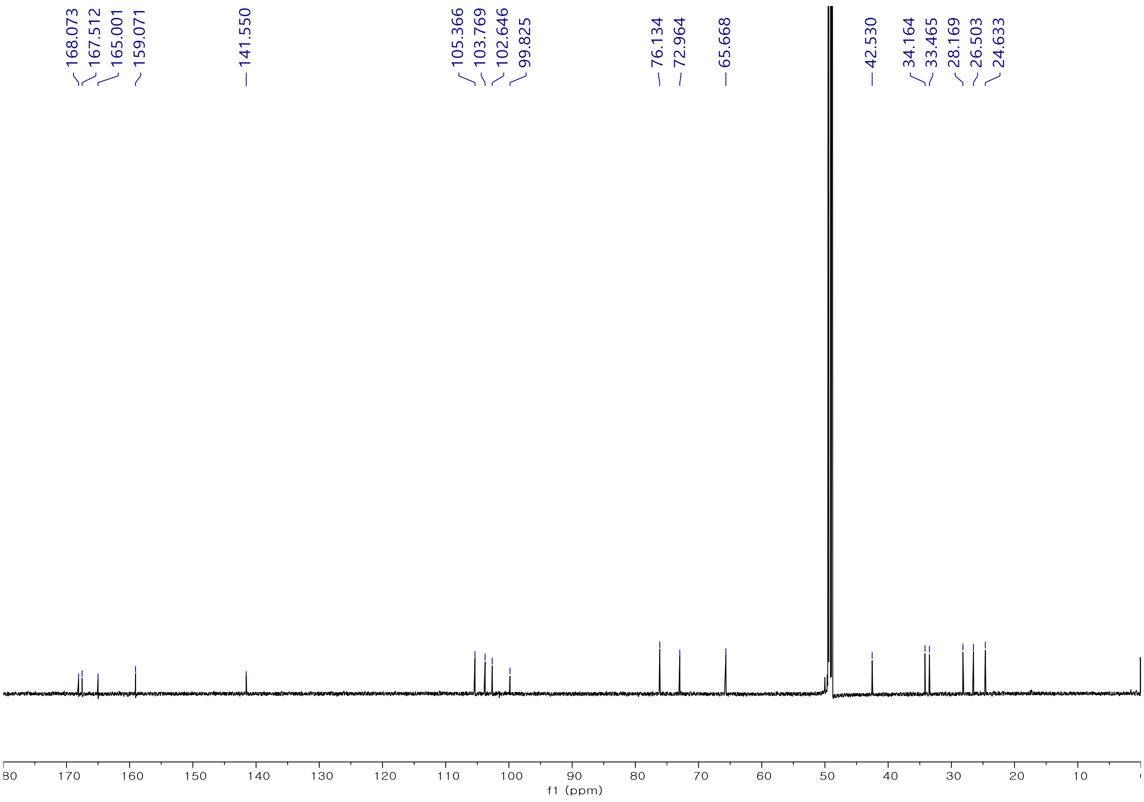

Figure S19. ^{1}H-^{1}H COSY spectrum (500 MHz, CD_3OD) of (**2**)

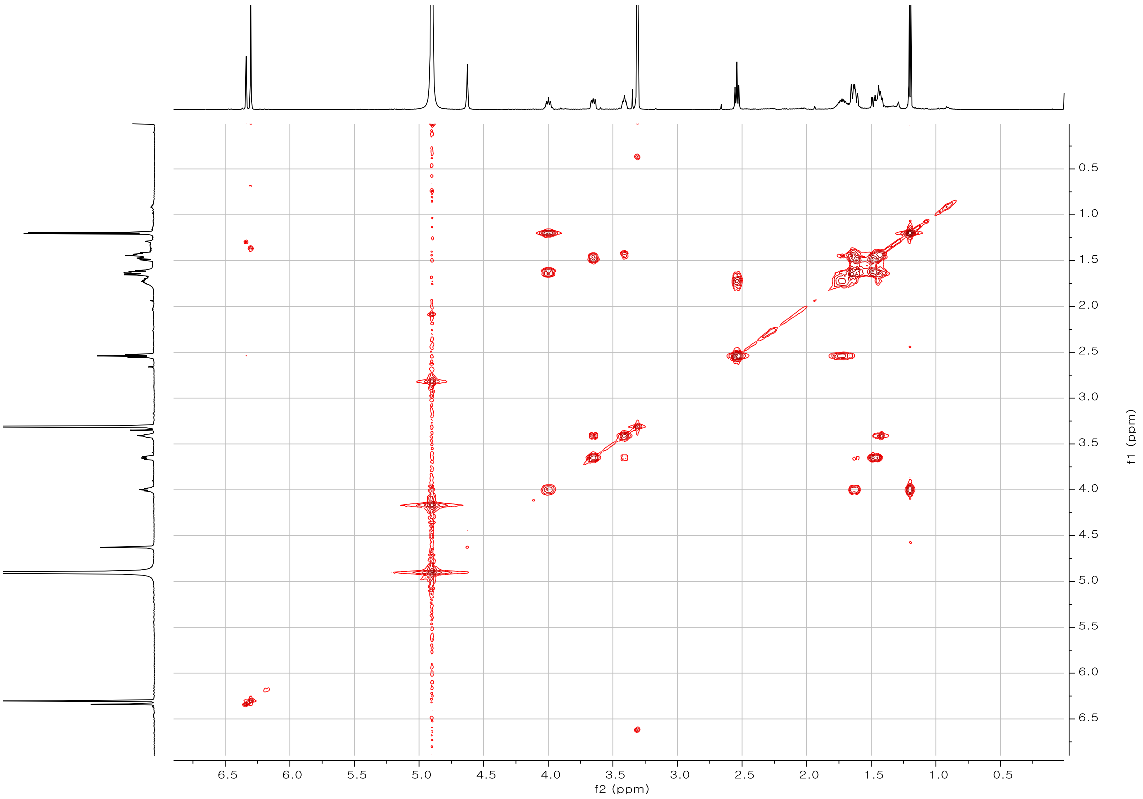

Figure S20. HSQC spectrum (500 MHz, CD_3OD) of (**2**)

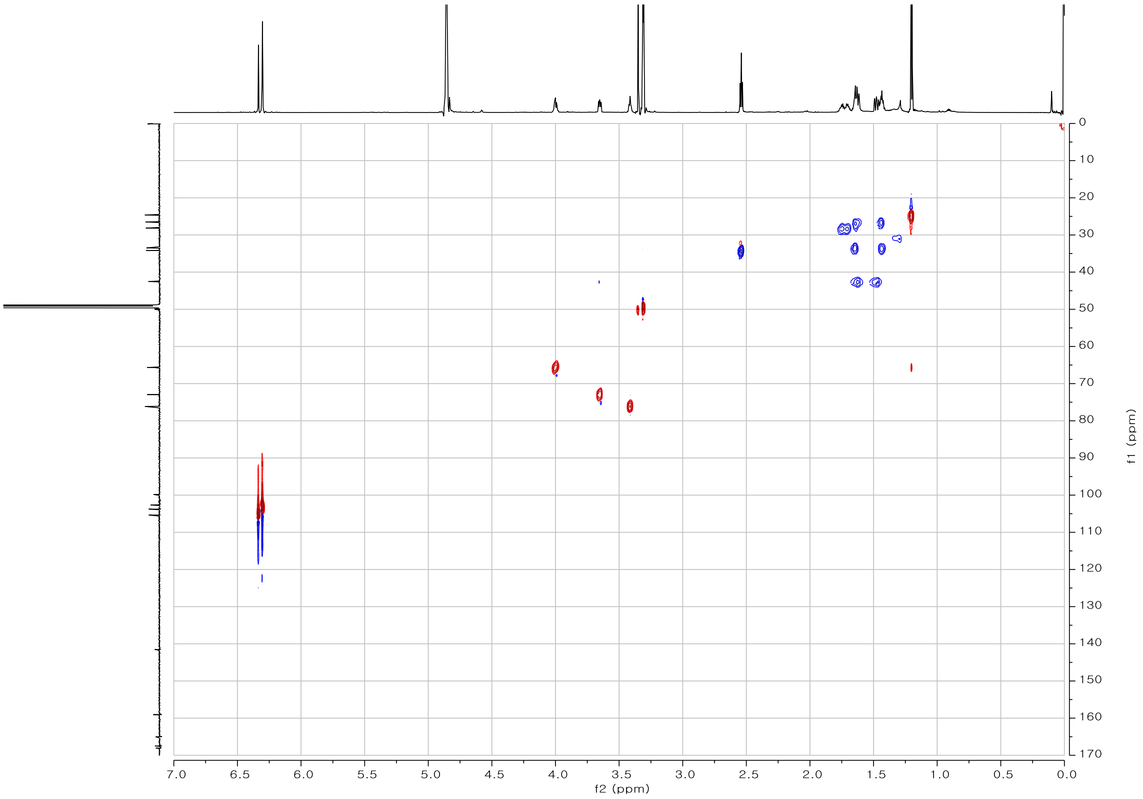

Figure S21. HMBC spectrum (500 MHz, CD_3OD) of (**2**)

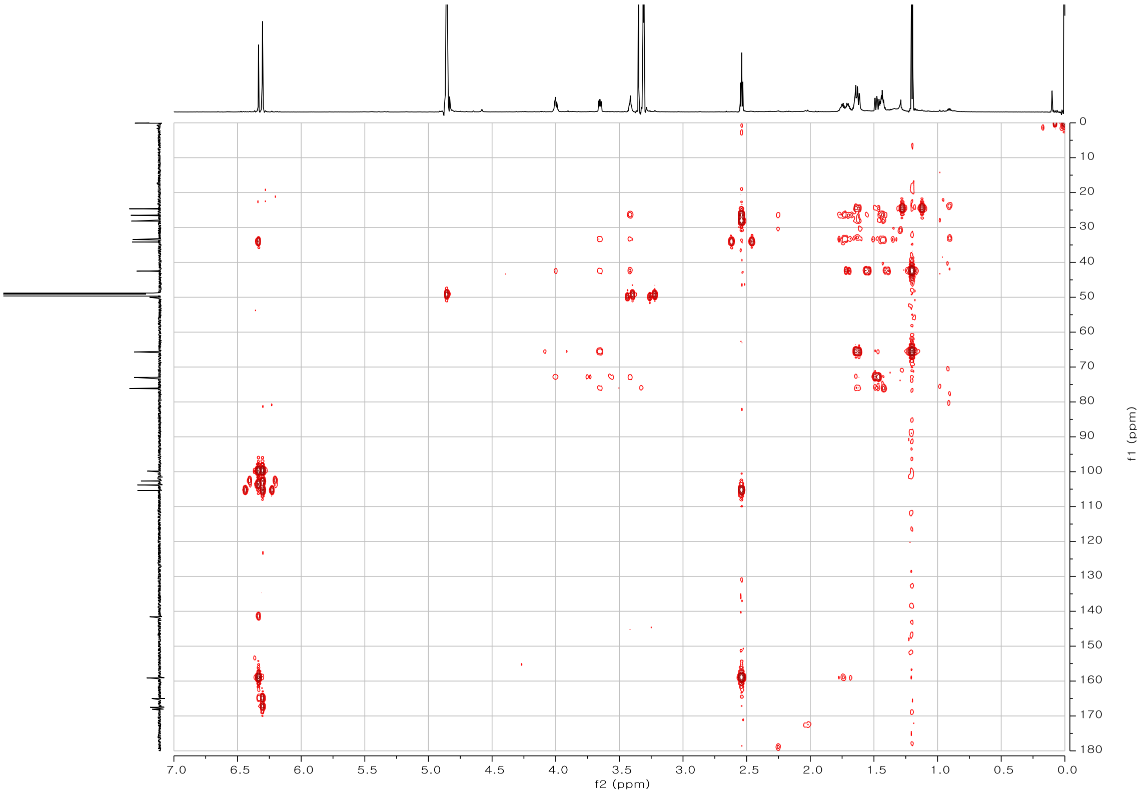

Figure S22. HRESIMS spectrum of (**2**)

Compound Spectrum SmartFormula Report

Analysis Info

Analysis Name	D:\Data\Data\2019\DSWU\SSH\BSH06\BSH006.d	Acquisition Date	6/27/2019 4:42:00 PM
Method	Tune_pos_Standard_100_600_190102.d.m	Operator	lee
Sample Name	BSH06_9H4E	Instrument	compact 8255754.10024
Comment			

Acquisition Parameter

Source Type	ESI	Ion Polarity	Positive	Set Nebulizer	0.4 Bar
Focus	Active	Set Capillary	4000 V	Set Dry Heater	200 °C
Scan Begin	100 m/z	Set End Plate Offset	-500 V	Set Dry Gas	4.0 l/min
Scan End	600 m/z	Set Charging Voltage	2000 V	Set Divert Valve	Source
		Set Corona	0 nA	Set APCI Heater	0 °C

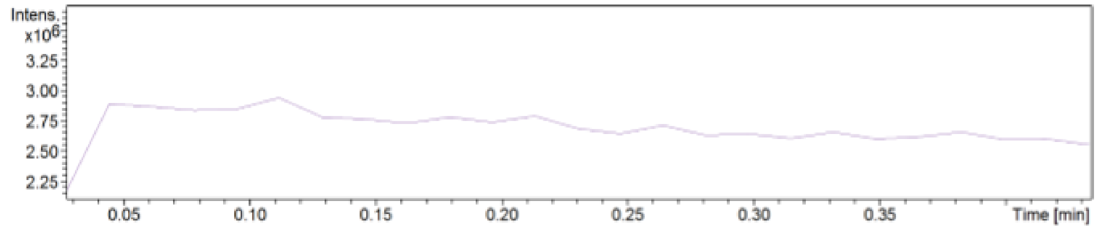

#	RT [min]	Area	Int. Type	I	S/N	Chromatogram	Max. m/z	FWHM [min]
n.a.	0.2	n.a.	Average spectrum	n.a.	n.a.	n.a.	375.1417	n.a.

+MS, 0.1-0.3min #8-17

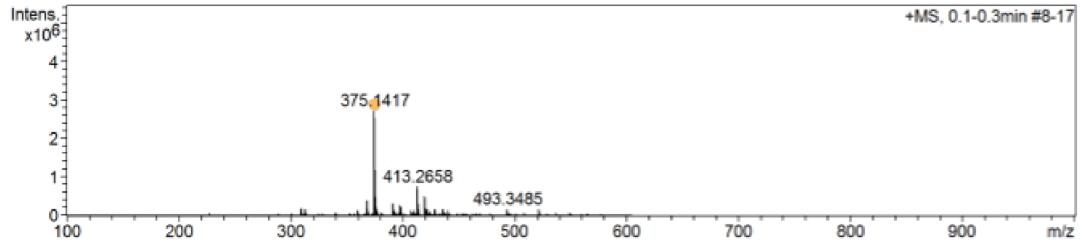

Meas. m/z	#	Ion Formula	m/z	err [ppm]	mSigma	# Sigma	Score	rdb	e⁻ Conf	N-Rule
375.1417	1	C16H19N6O5	375.1411	1.5	3.9	1	100.00	10.5	even	ok
	2	C15H23N2O9	375.1398	-5.0	8.6	2	54.19	5.5	even	ok
	3	C12H15N12O3	375.1385	8.6	10.9	3	11.94	11.5	even	ok
	4	C20H23O7	375.1438	5.7	14.9	4	39.35	9.5	even	ok
	5	C17H15N10O	375.1425	2.1	17.5	5	68.13	15.5	even	ok
	6	C21H19N4O3	375.1452	9.3	26.3	6	8.88	14.5	even	ok
	7	C27H19O2	375.1380	9.9	51.8	7	3.37	18.5	even	ok
	1	C18H24NaO7	375.1414	-0.7	4.0	1	100.00	6.5	even	ok
	2	C15H16N10NaO	375.1401	-4.3	7.3	2	33.23	12.5	even	ok
	3	C14H20N6NaO5	375.1387	-7.9	9.3	3	10.70	7.5	even	ok
	4	C19H20N4NaO3	375.1428	-2.9	17.9	4	38.16	11.5	even	ok

Figure S23. ^{1}H NMR spectrum (500 MHz, CD_3OD) of (**3**)

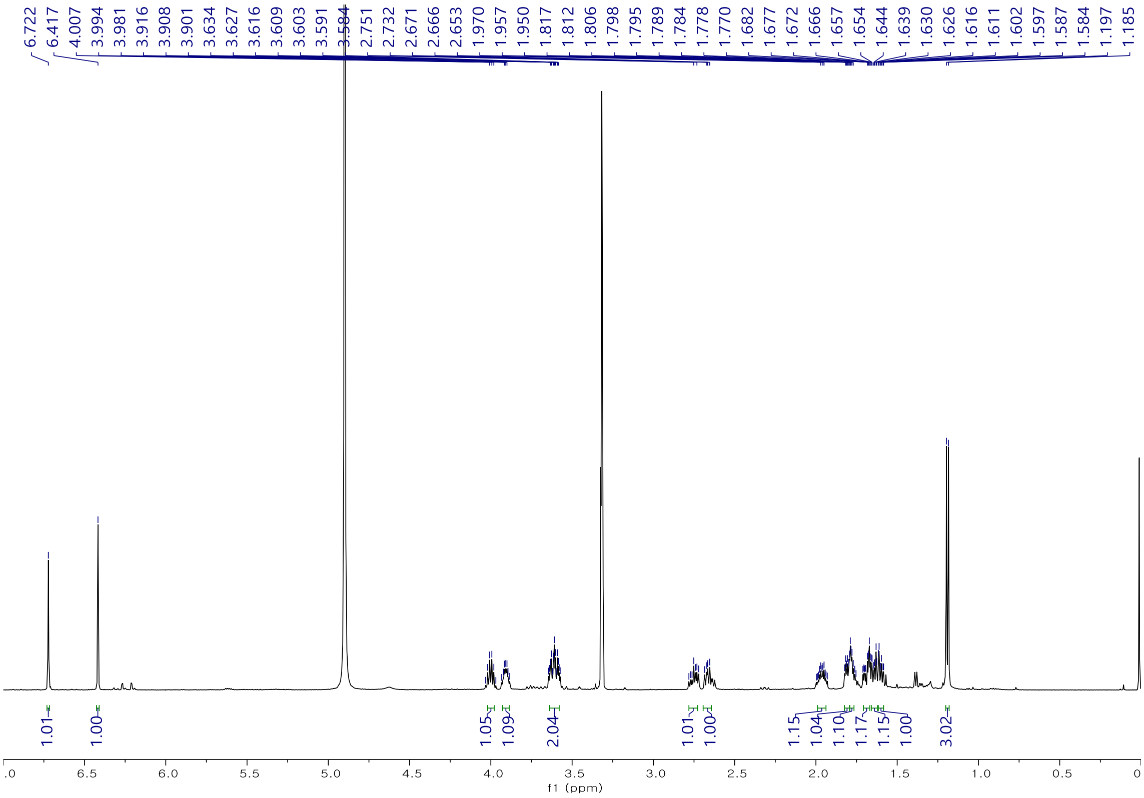

Figure S24. ^{13}C NMR spectrum (125 MHz, CD_3OD) of (**3**)

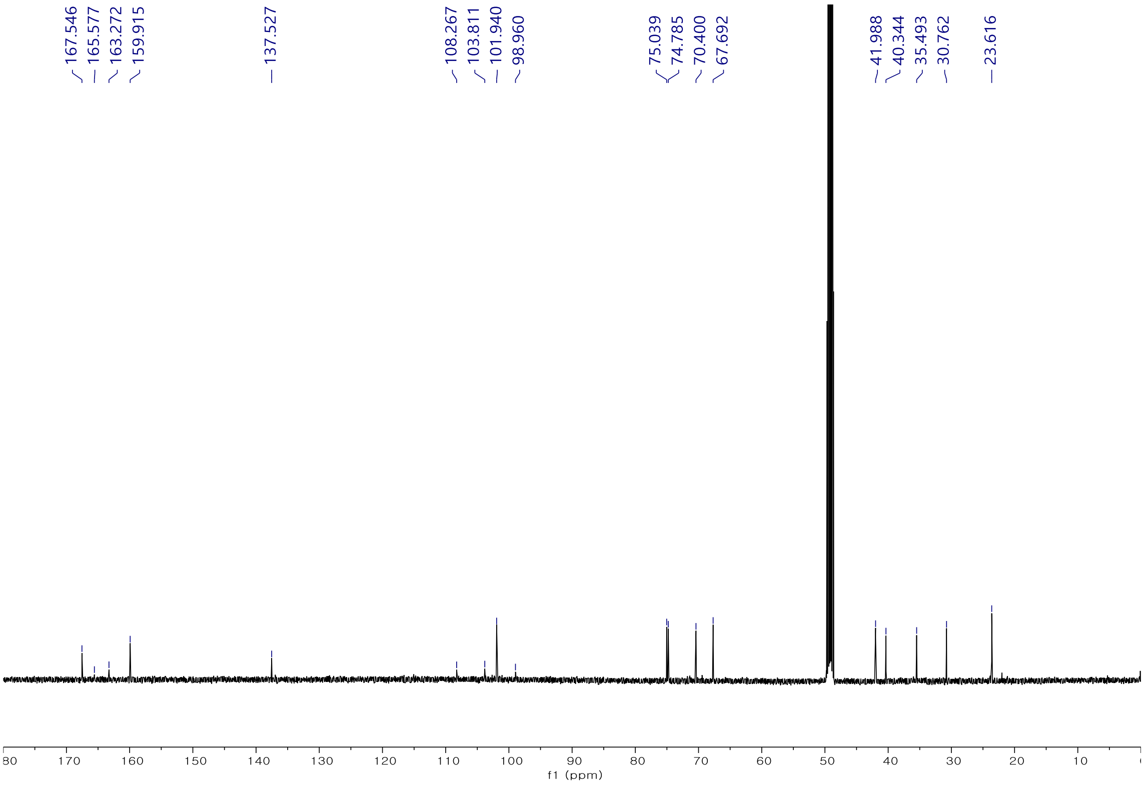

Figure S25. ^{1}H-^{1}H COSY spectrum (500 MHz, CD_3OD) of (**3**)

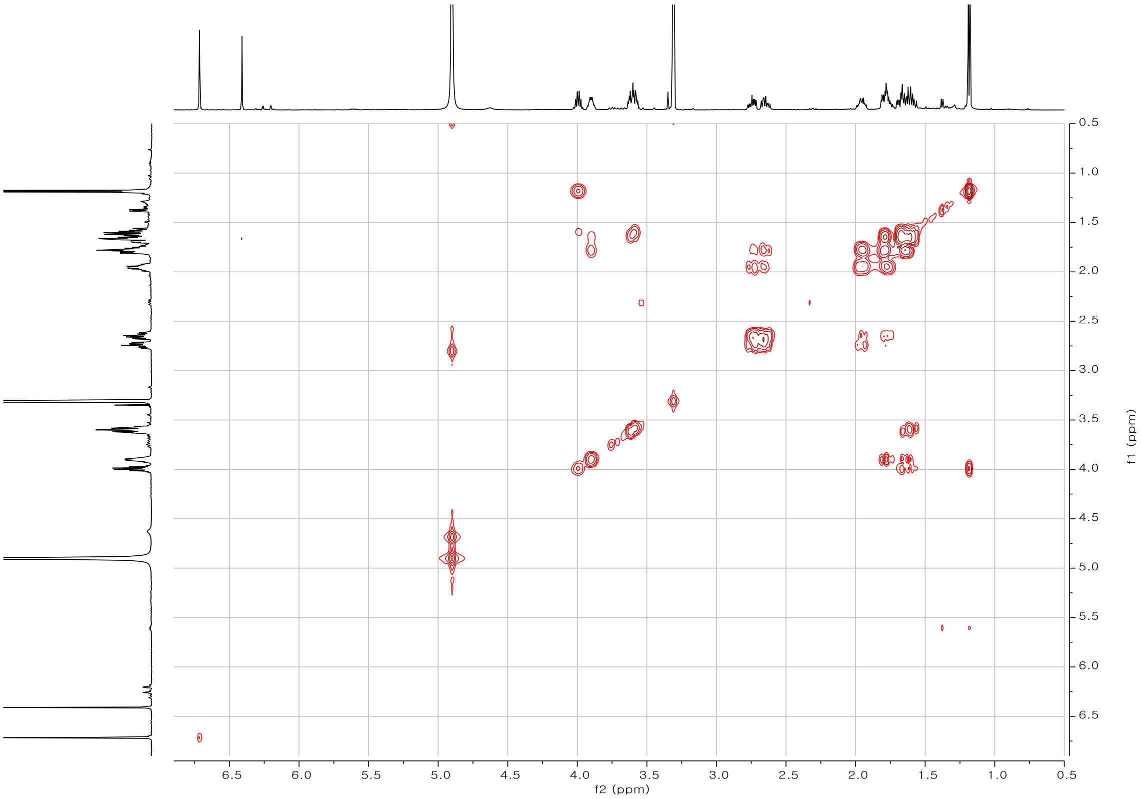

Figure S26. HSQC spectrum (500 MHz, CD_3OD) of (**3**)

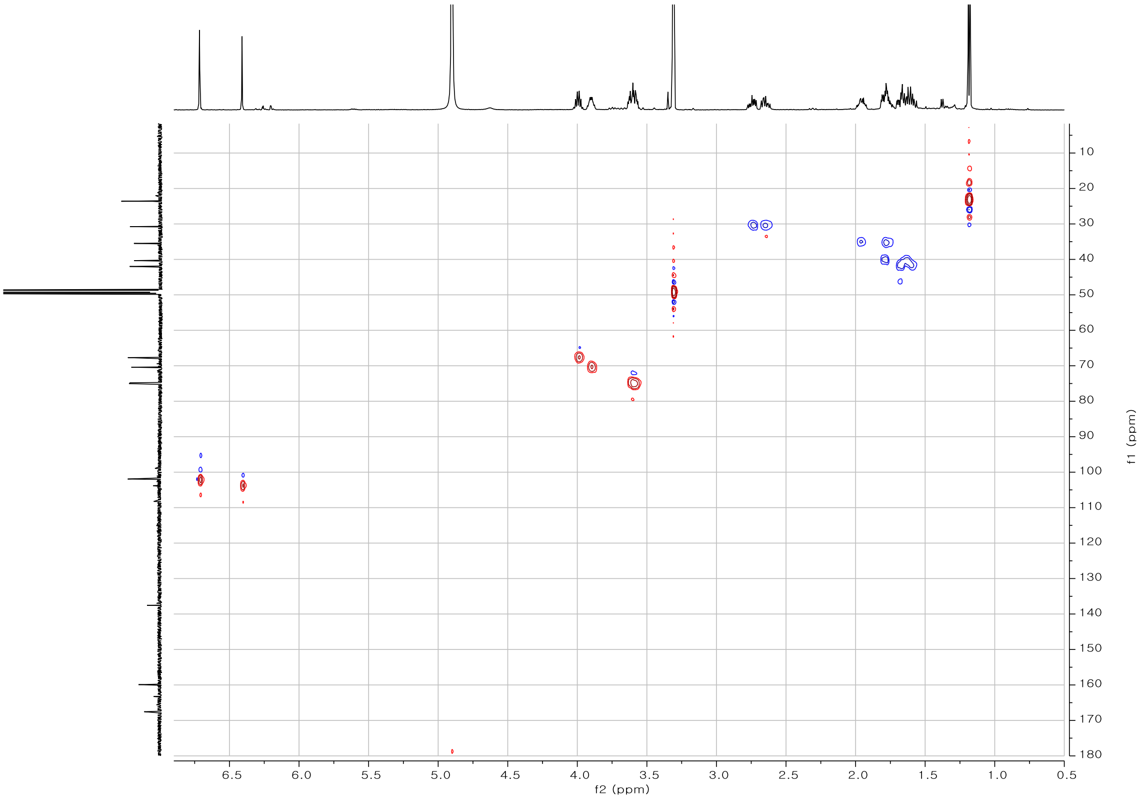

Figure S27. HMBC spectrum (500 MHz, CD_3OD) of (**3**)

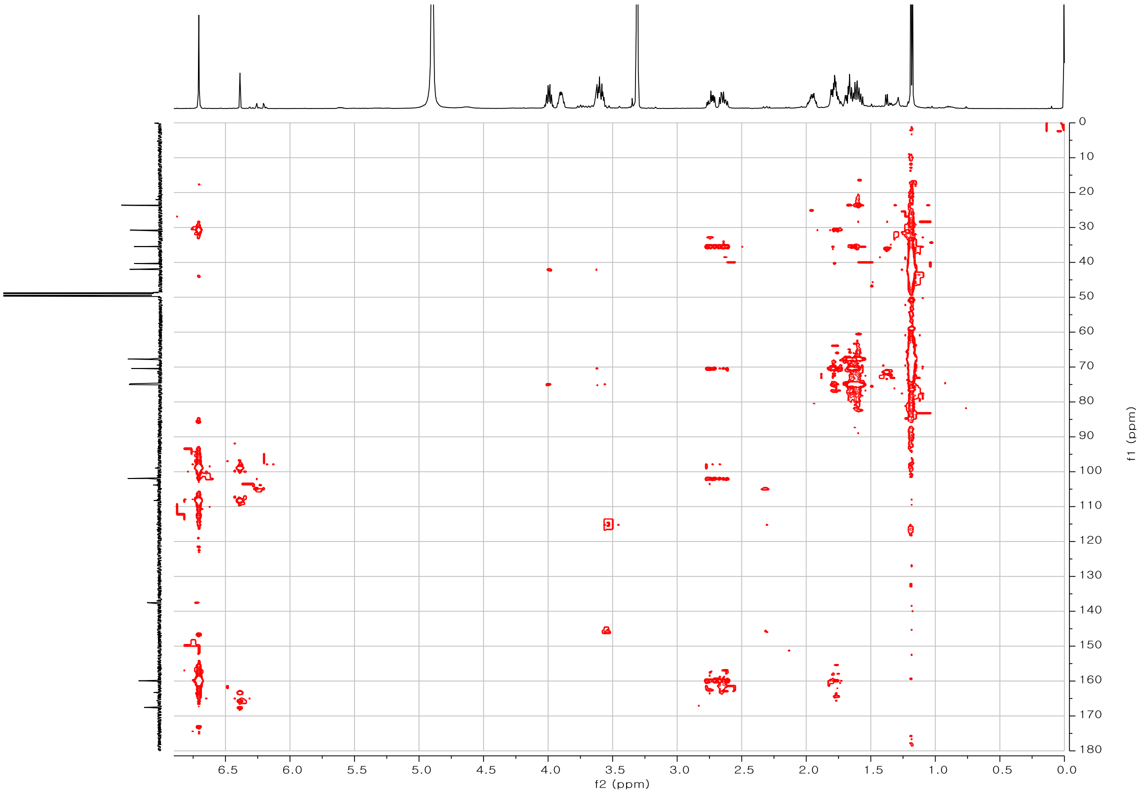

Figure S28. HETLOC spectrum (800 MHz, CD_3OD) of (**3**)

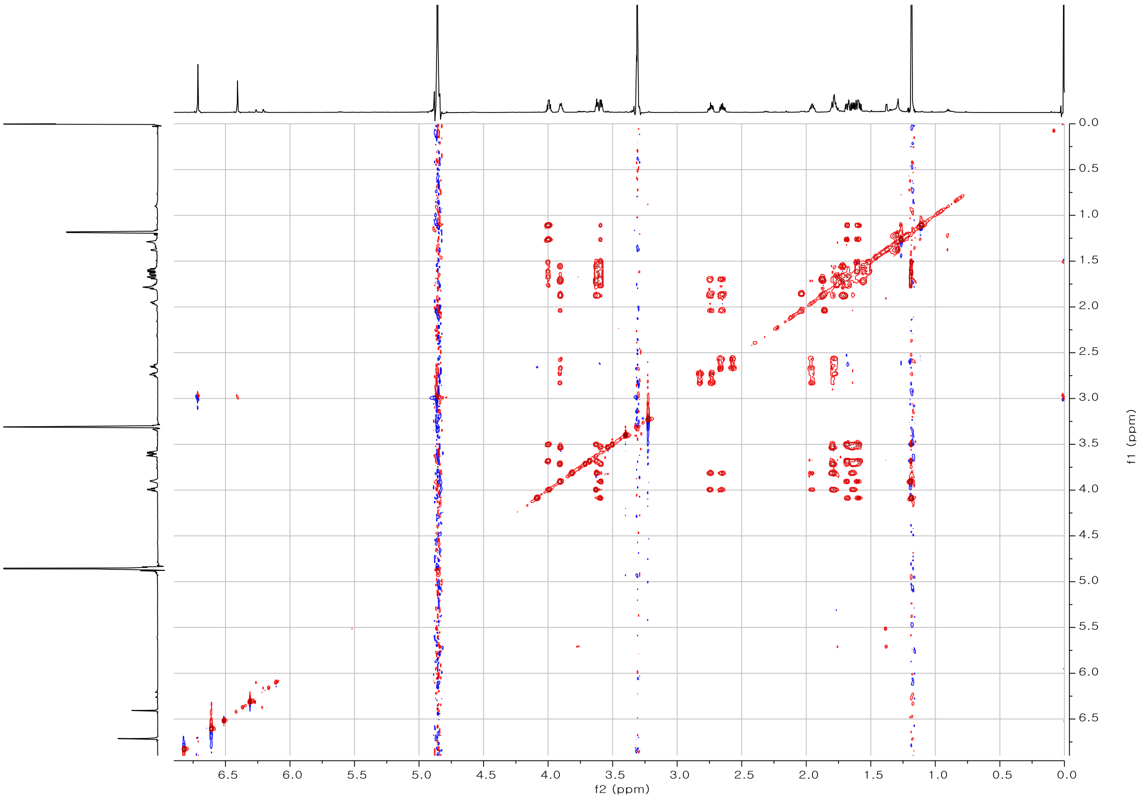

Figure S29. ^{1}H NMR spectrum (500 MHz, CD_3OD) of acetonide product (**3a**)

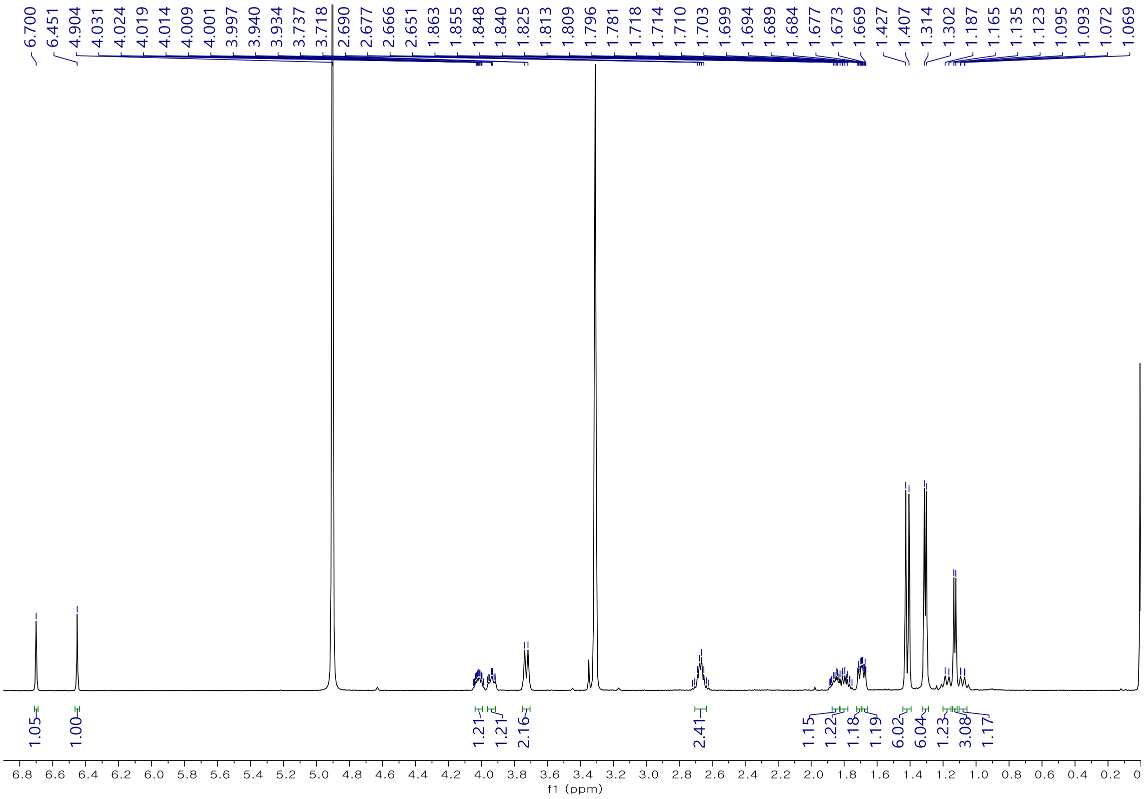

Figure S30. ^{13}C NMR spectrum (125 MHz, CD_3OD) of acetonide product (**3a**)

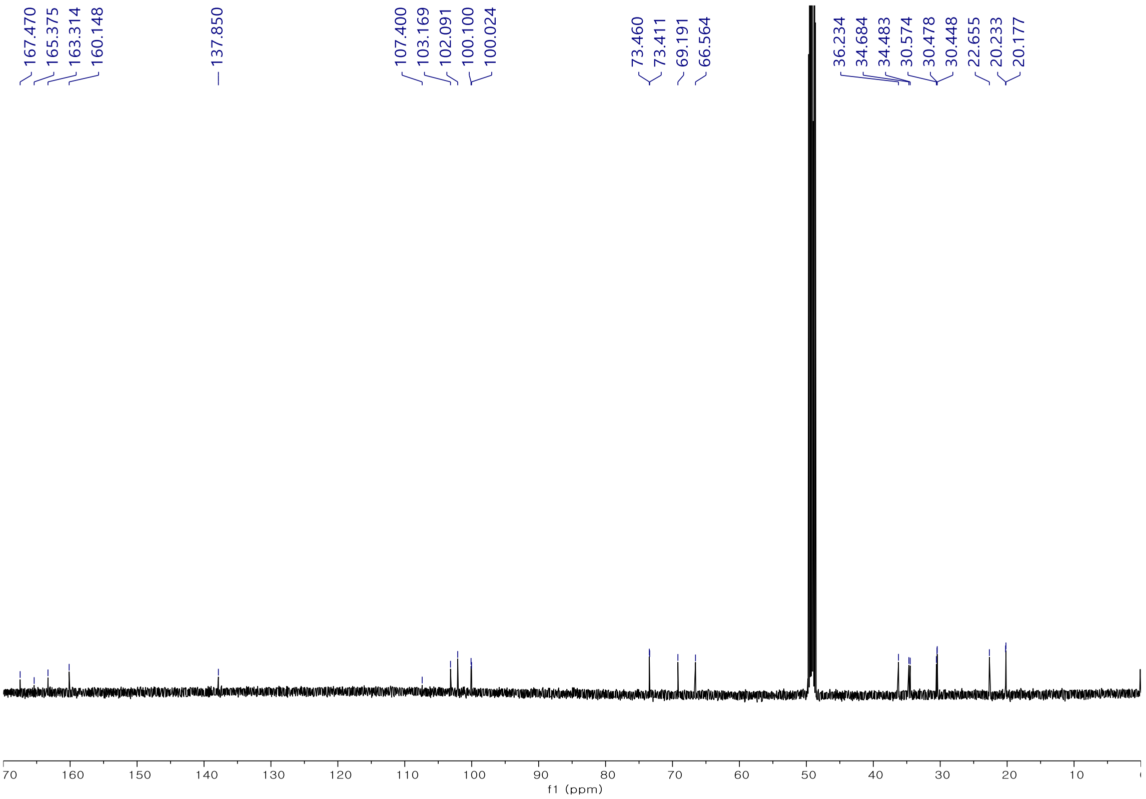

Figure S31. HMBC spectrum (500 MHz, CD_3OD) of acetonide product (**3a**)

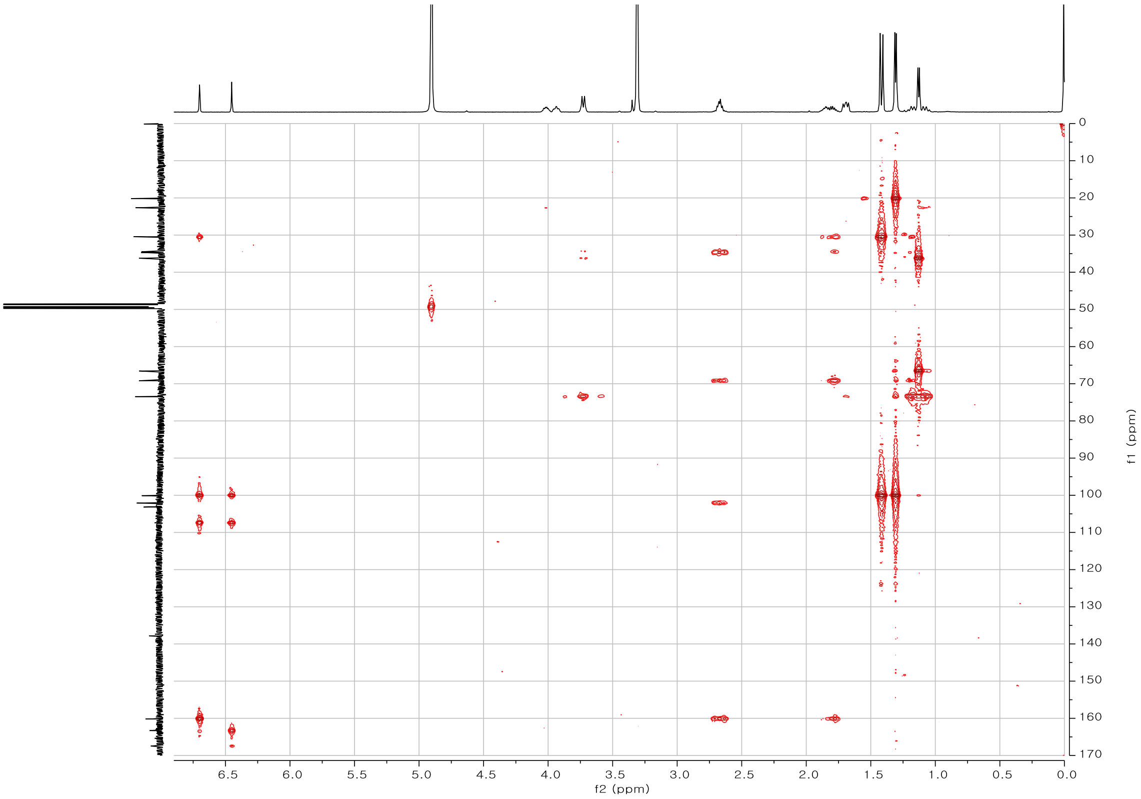

Figure S32. HRESIMS spectrum of (**3**)

Compound Spectrum SmartFormula Report

Analysis Info

Analysis Name	D:\Data\Data\2019\DSWU\SSH\BSH06\BSH003.d	Acquisition Date	6/27/2019 11:45:43 AM
Method	Tune_pos_Standard_100_600_190102.d.m	Operator	lee
Sample Name	BSH03_9G3D2	Instrument	compact 8255754.10024
Comment			

Acquisition Parameter

Source Type	ESI	Ion Polarity	Positive	Set Nebulizer	0.4 Bar
Focus	Active	Set Capillary	4000 V	Set Dry Heater	200 °C
Scan Begin	100 m/z	Set End Plate Offset	-500 V	Set Dry Gas	4.0 l/min
Scan End	600 m/z	Set Charging Voltage	2000 V	Set Divert Valve	Source
		Set Corona	0 nA	Set APCI Heater	0 °C

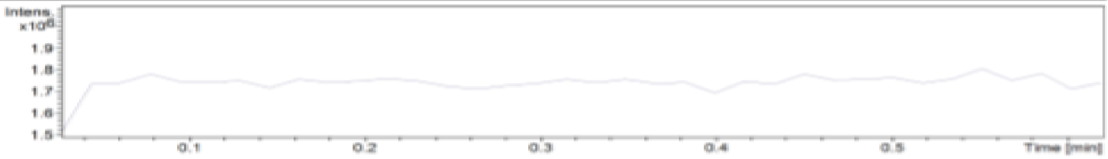

#	RT [min]	Area	Int. Type	I	S/N	Chromatogram	Max. m/z	FWHM [min]
n.a.	0.3	n.a.	Average spectrum	n.a.	n.a.	n.a.	425.1010	n.a.

+MS, 0.2-0.3min #11-19

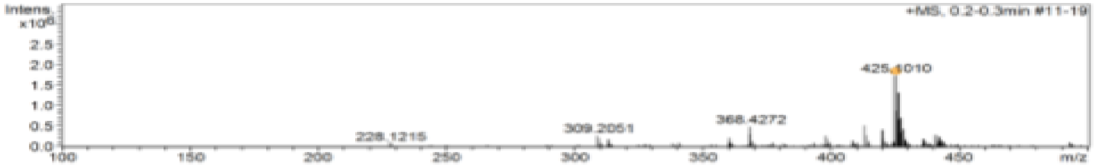

Meas. m/z	#	Ion Formula	m/z	err [ppm]	mSigma	# Sigma	Score	rdb	e⁻ Conf	N-Rule
425.1010	1	C20H23Cl2N2O4	425.1029	-4.5	230.4	1	100.00	9.5	even	ok
	2	C21H18ClN4O4	425.1011	0.2	243.4	2	56.35	14.5	even	ok
	3	C20H22ClO8	425.0998	-2.9	254.1	3	10.31	9.5	even	ok
	4	C17H14ClN10O2	425.0984	-6.1	254.7	4	3.54	15.5	even	ok
	5	C16H19Cl2N8O2	425.1003	-1.8	256.2	5	13.03	10.5	even	ok
	6	C14H27Cl2O10	425.0976	-8.1	256.5	6	1.80	0.5	even	ok
	7	C16H18ClN6O6	425.0971	9.3	265.3	7	0.29	10.5	even	ok
	8	C15H23Cl2N4O6	425.0989	5.0	266.5	8	1.78	5.5	even	ok
	9	C10H18ClN10O7	425.1043	-7.7	289.0	9	0.04	6.5	even	ok
	10	C9H22ClN6O11	425.1030	-4.6	299.7	10	0.04	1.5	even	ok
	11	C6H14ClN16O5	425.1016	-1.4	300.1	11	0.08	7.5	even	ok
	12	C19H28Cl3O4	425.1048	-8.8	303.0	12	0.01	4.5	even	ok
	13	C15H24Cl3N6O2	425.1021	2.5	305.6	13	0.04	5.5	even	ok
	14	C14H28Cl3N2O6	425.1007	0.7	313.1	14	0.02	0.5	even	ok
	15	C10H24Cl3N8O4	425.0981	7.0	316.8	15	0.00	1.5	even	ok
	16	C14H29Cl4N4O2	425.1039	-6.8	337.4	16	0.00	0.5	even	ok
	1	C23H24Cl2NaO2	425.1046	-8.3	221.8	1	71.68	10.5	even	ok
	2	C24H19ClN2NaO2	425.1027	4.0	231.9	2	100.00	15.5	even	ok
	3	C19H19ClN4NaO4	425.0987	5.5	253.8	3	6.84	11.5	even	ok

BSH003.d
Bruker Compass DataAnalysis 4.1 printed: 10/29/2019 10:46:29 AM by: lee Page 1 of 2

Compound Spectrum SmartFormula Report

Meas. m/z	#	Ion Formula	m/z	err [ppm]	mSigma	# Sigma	Score	rdb	e⁻ Conf	N-Rule
	4	C18H24Cl2N2NaO4	425.1005	1.2	256.6	4	20.06	6.5	even	ok
	5	C14H20Cl2N8NaO2	425.0978	-7.5	264.3	5	1.22	7.5	even	ok
	6	C18H23ClNaO8	425.0974	8.6	264.5	6	0.60	6.5	even	ok
	7	C12H23ClN4NaO9	425.1046	8.4	288.2	7	0.04	2.5	even	ok
	8	C9H15ClN14NaO3	425.1032	5.2	288.7	8	0.15	8.5	even	ok
	9	C8H19ClN10NaO7	425.1019	2.0	299.3	9	0.10	3.5	even	ok
	10	C17H29Cl3NaO4	425.1024	-3.2	306.8	10	0.04	1.5	even	ok
	11	C13H25Cl3N6NaO2	425.0997	-3.2	309.9	11	0.03	2.5	even	ok

Figure S33. ^{1}H NMR spectrum (500 MHz, CD_3OD) of (**4**)

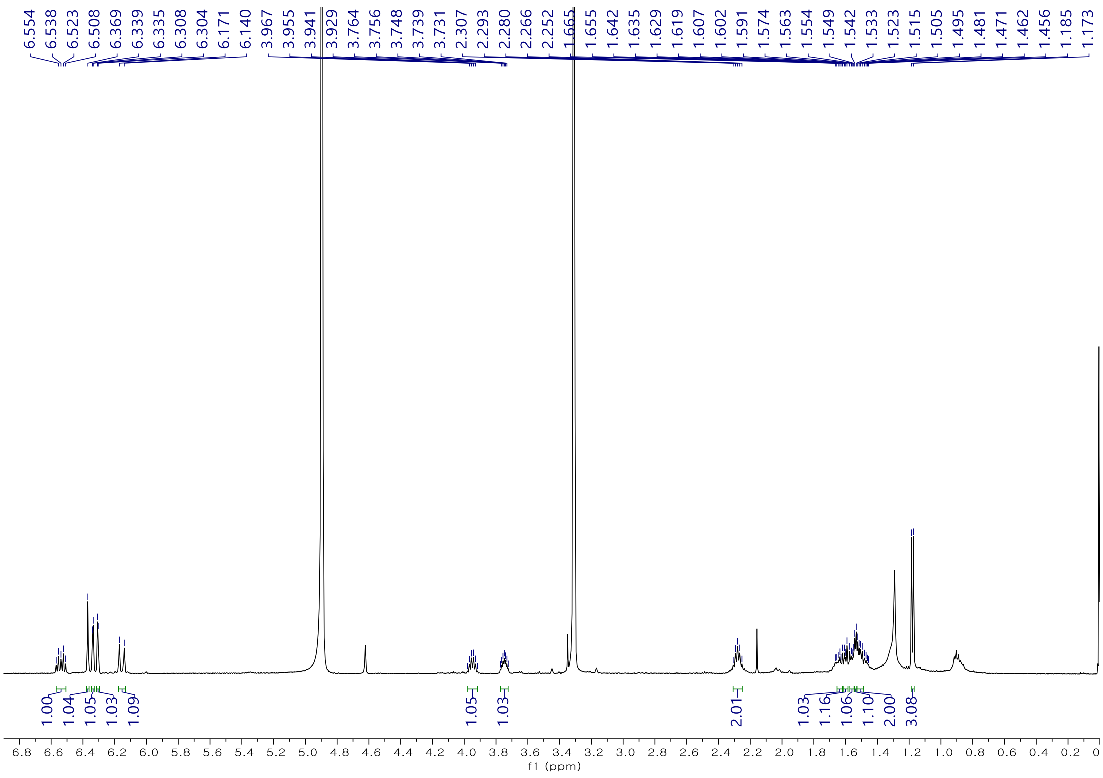

Figure S34. ^{13}C NMR spectrum (125 MHz, CD_3OD) of (**4**)

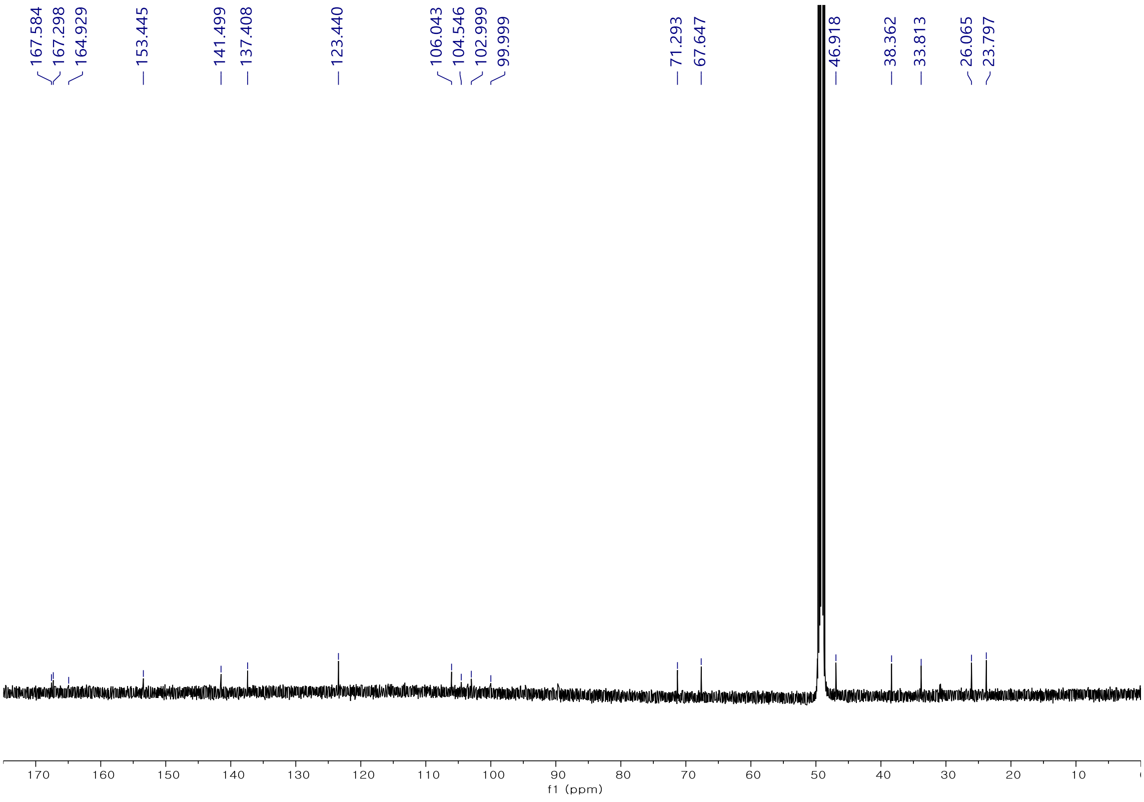

Figure S35. ^1H-^1H COSY spectrum (500 MHz, CD_3OD) of (**4**)

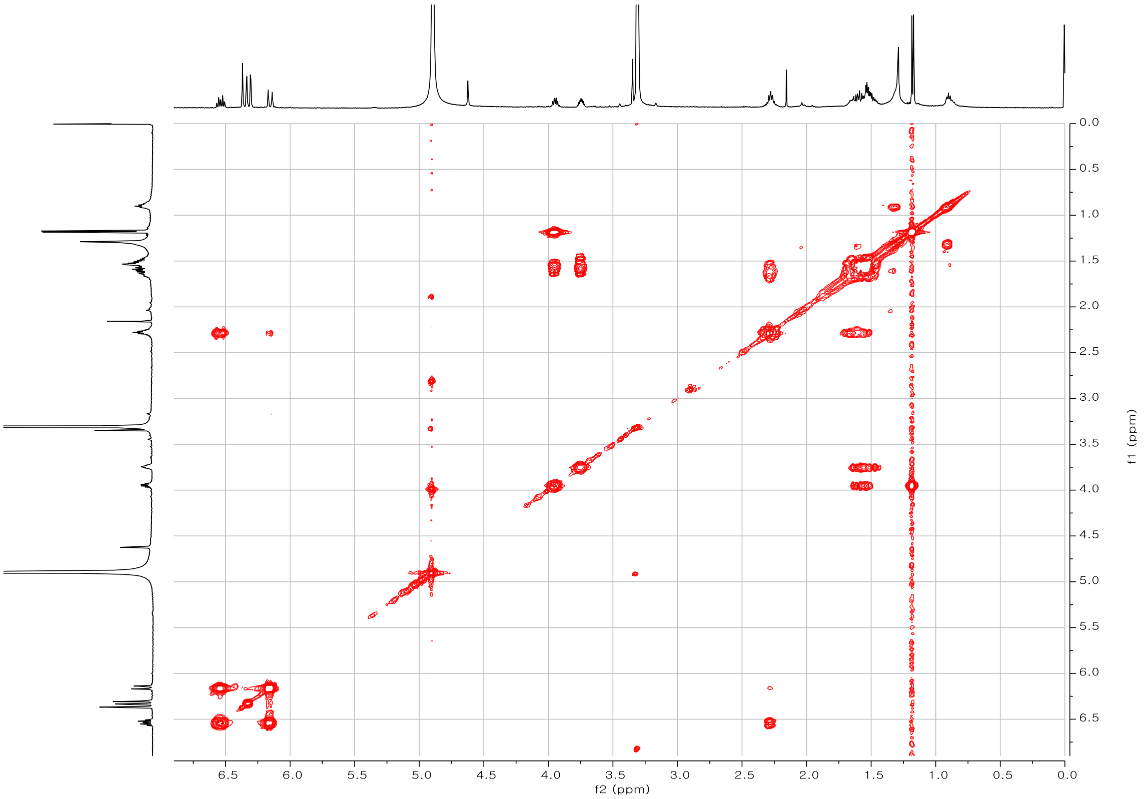

Figure S36. HSQC spectrum (500 MHz, CD_3OD) of (**4**)

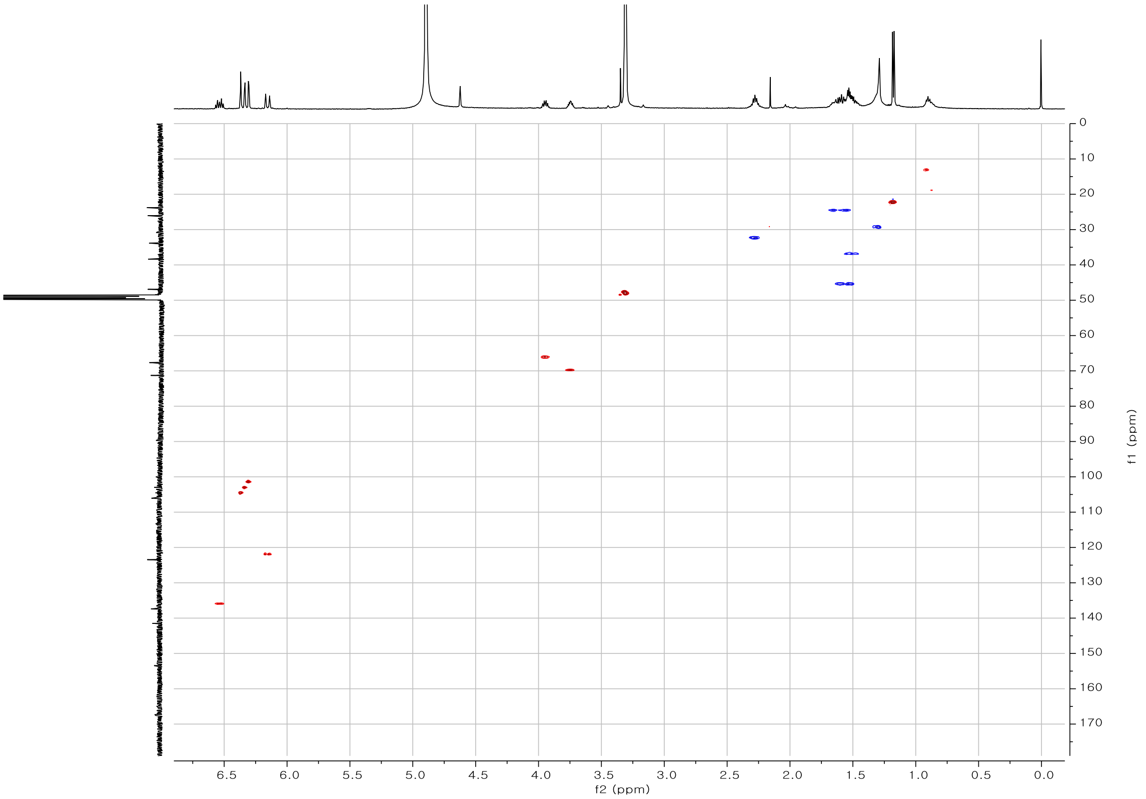

Figure S37. HMBC spectrum (500 MHz, CD_3OD) of (**4**)

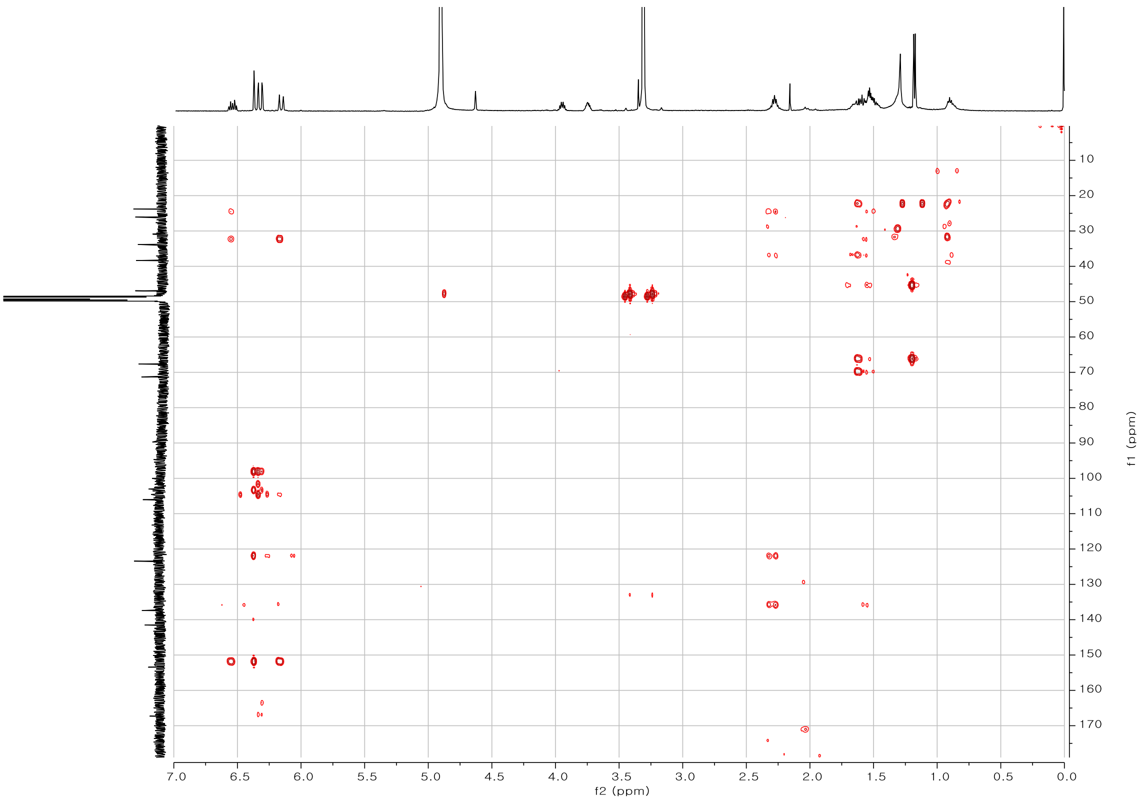

Figure S38. HRESIMS spectrum of (**4**)

Compound Spectrum SmartFormula Report

Analysis Info

Analysis Name	D:\Data\Data\2019\DSWU\SSH\BSH06\BSH009.d	Acquisition Date	6/27/2019 4:58:32 PM
Method	Tune_pos_Standard_100_600_190102.d.m	Operator	lee
Sample Name	BSH09_8F5D1	Instrument	compact 8255754.10024
Comment			

Acquisition Parameter

Source Type	ESI	Ion Polarity	Positive	Set Nebulizer	0.4 Bar
Focus	Active	Set Capillary	4000 V	Set Dry Heater	200 °C
Scan Begin	100 m/z	Set End Plate Offset	-500 V	Set Dry Gas	4.0 l/min
Scan End	600 m/z	Set Charging Voltage	2000 V	Set Divert Valve	Source
		Set Corona	0 nA	Set APCI Heater	0 °C

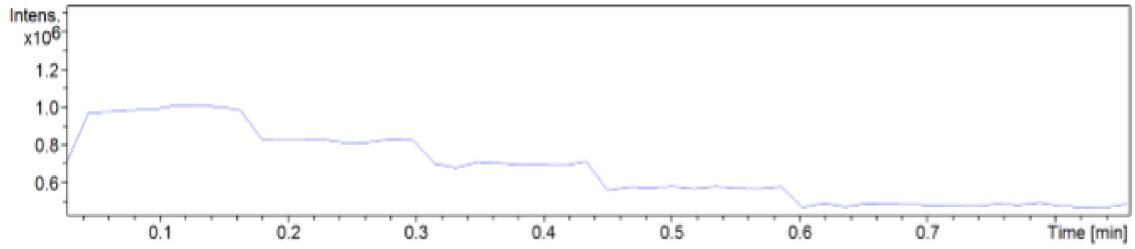

#	RT [min]	Area	Int. Type	I	S/N	Chromatogram	Max. m/z	FWHM [min]
n.a.	0.6	n.a.	Average spectrum	n.a.	n.a.	n.a.	357.1312	n.a.

+MS, 0.5-0.8min #30-44

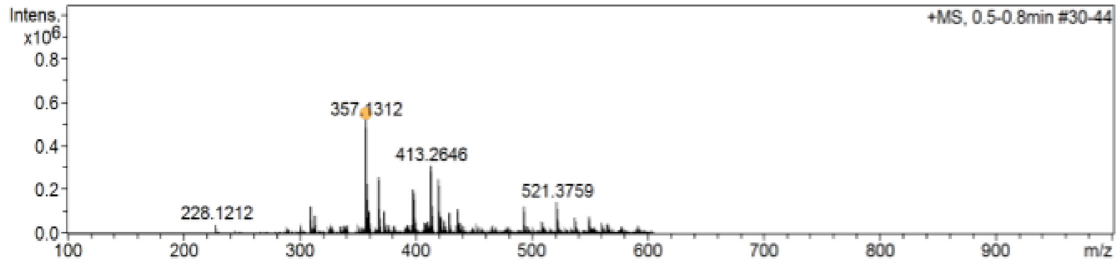

Meas. m/z	#	Ion Formula	m/z	err [ppm]	mSigma	# Sigma	Score	rdb	e⁻ Conf	N-Rule
357.1312	1	C27H17O	357.1274	10.7	17.9	1	33.92	19.5	even	ok
	2	C21H17N4O2	357.1346	-9.4	48.2	2	19.72	15.5	even	ok
	3	C20H21O6	357.1333	5.7	52.3	3	78.03	10.5	even	ok
	4	C17H13N10	357.1319	1.9	62.2	4	100.00	16.5	even	ok
	5	C16H17N6O4	357.1306	1.8	74.2	5	65.96	11.5	even	ok
	6	C15H21N2O8	357.1292	-5.6	75.1	6	36.53	6.5	even	ok
	7	C12H13N12O2	357.1279	9.3	88.0	7	4.80	12.5	even	ok
	1	C19H18N4NaO2	357.1322	2.7	60.7	1	60.24	12.5	even	ok
	2	C18H22NaO6	357.1309	-1.0	63.2	2	100.00	7.5	even	ok
	3	C15H14N10Na	357.1295	4.8	74.7	3	22.54	13.5	even	ok
	4	C14H18N6NaO4	357.1282	-8.6	86.7	4	4.48	8.5	even	ok

Figure S39. ^{1}H NMR spectrum (500 MHz, CD_3OD) of (**5**)

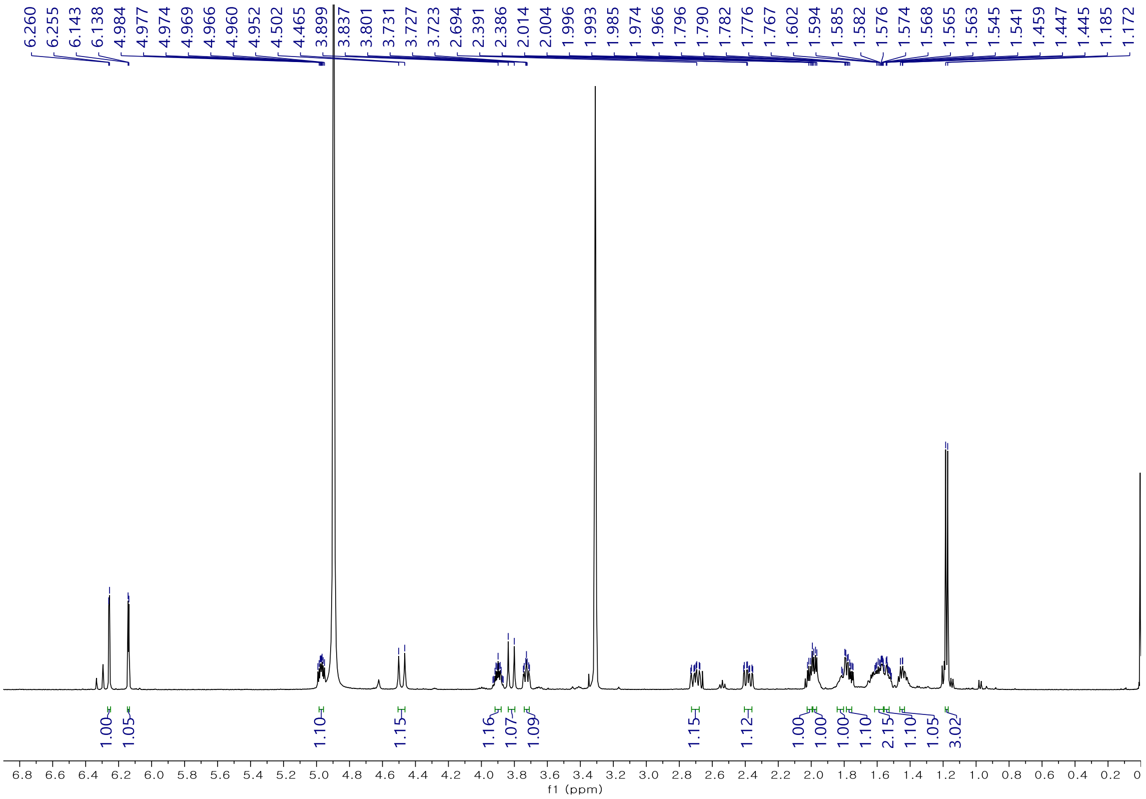

Figure S40. ^{13}C NMR spectrum (125 MHz, CD_3OD) of (**5**)

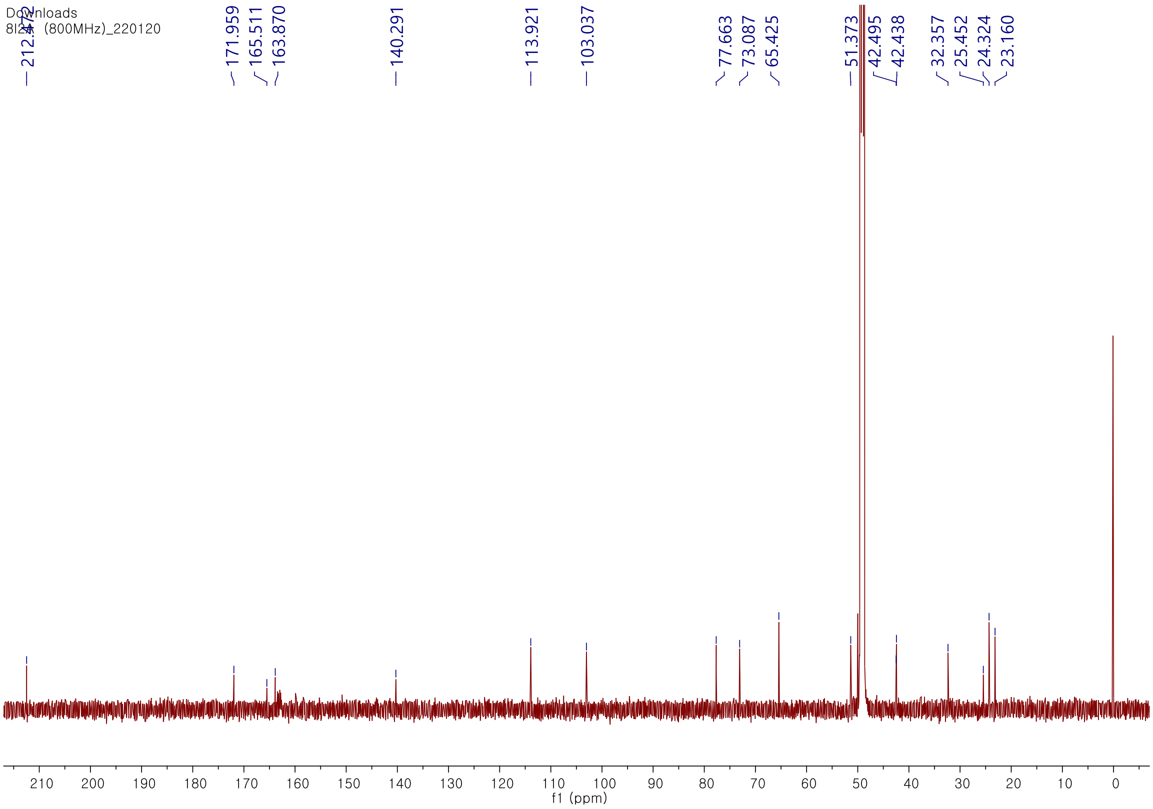

Figure S41. ^{1}H-^{1}H COSY spectrum (500 MHz, CD_3OD) of (**5**)

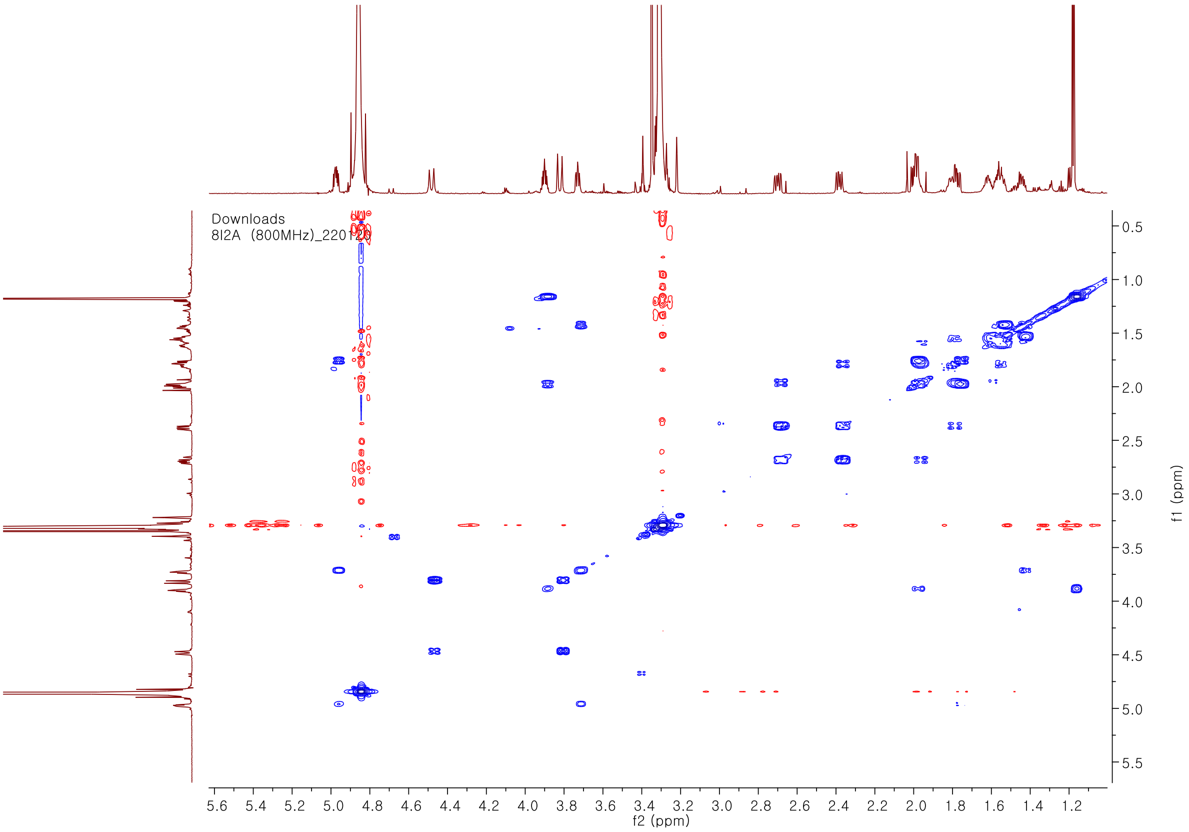

Figure S42. HSQC spectrum (500 MHz, CD_3OD) of (**5**)

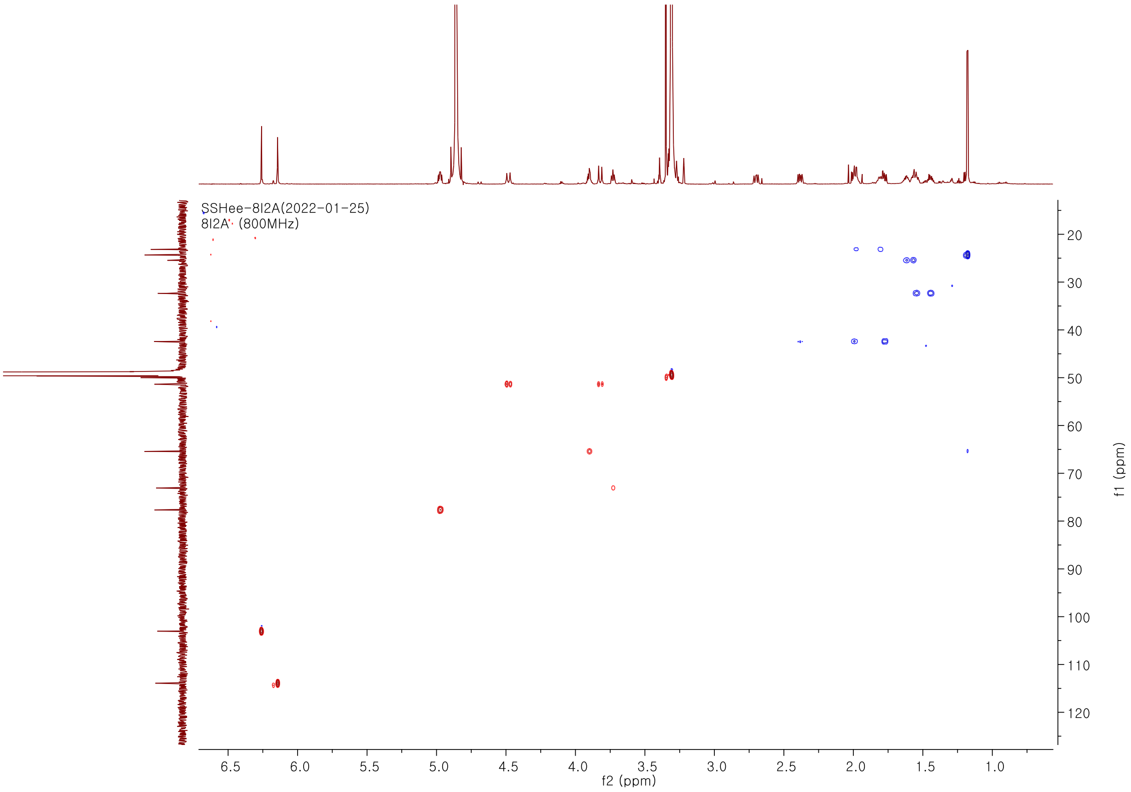

Figure S43. HMBC spectrum (500 MHz, CD_3OD) of (**5**)

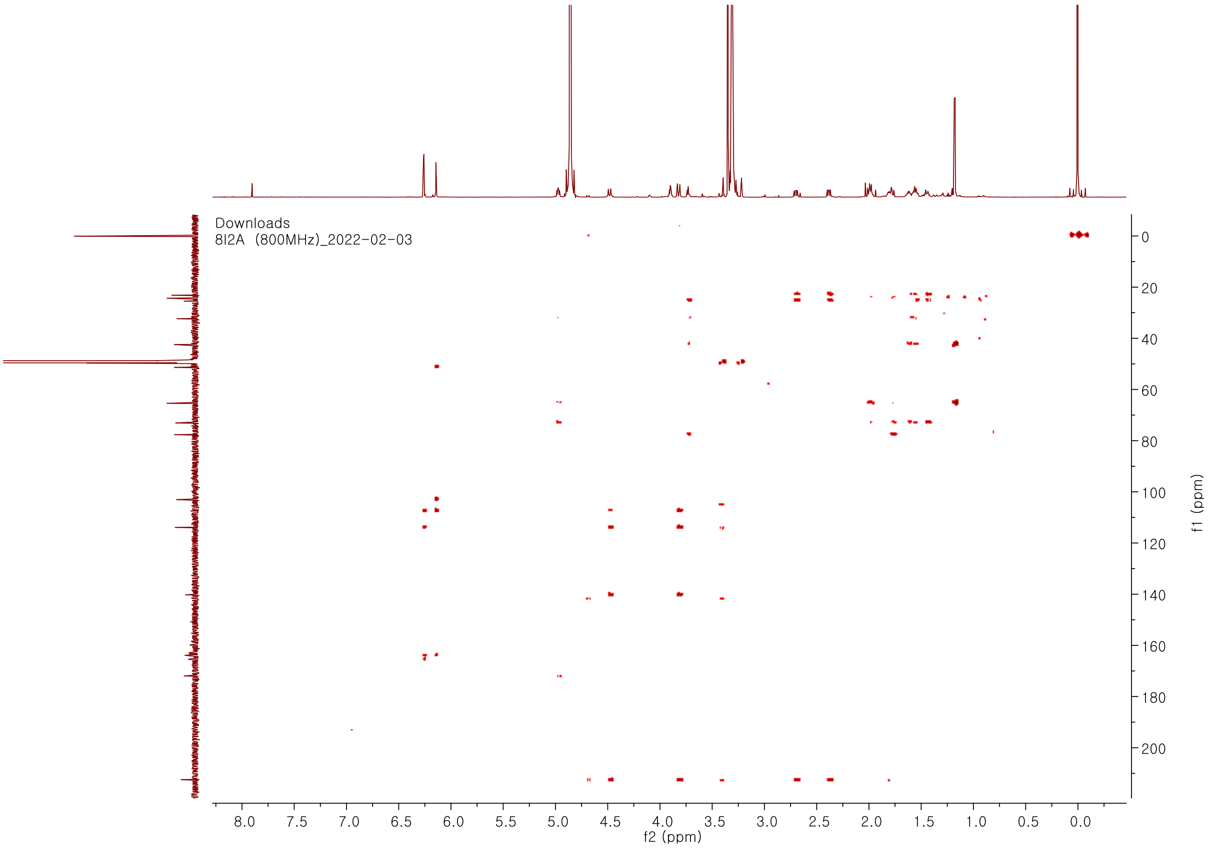

Figure S44. HRESIMS spectrum of (**5**)

Compound Spectrum SmartFormula Report

Analysis Info

Analysis Name	D:\Data\Data\2019\DSWU\SSH\BSH06\BSH010.d	Acquisition Date	6/27/2019 5:01:28 PM
Method	Tune_pos_Standard_100_600_190102.d.m	Operator	lee
Sample Name	BSH10_8I2A	Instrument	compact 8255754.10024
Comment			

Acquisition Parameter

Source Type	ESI	Ion Polarity	Positive	Set Nebulizer	0.4 Bar
Focus	Active	Set Capillary	4000 V	Set Dry Heater	200 °C
Scan Begin	100 m/z	Set End Plate Offset	-500 V	Set Dry Gas	4.0 l/min
Scan End	600 m/z	Set Charging Voltage	2000 V	Set Divert Valve	Source
		Set Corona	0 nA	Set APCI Heater	0 °C

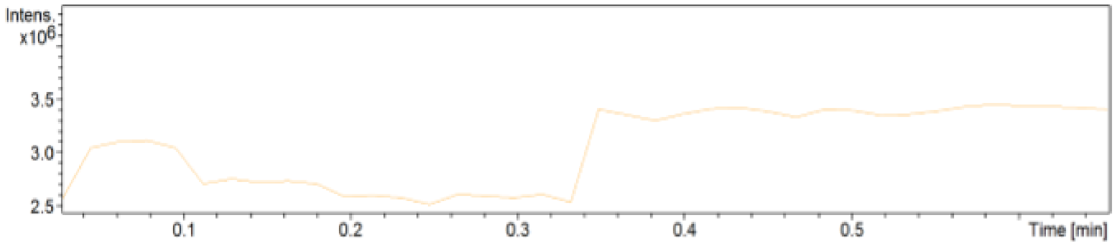

#	RT [min]	Area	Int. Type	I	S/N	Chromatogram	Max. m/z	FWHM [min]
n.a.	0.4	n.a.	Average spectrum	n.a.	n.a.	n.a.	375.1419	n.a.

+MS, 0.3-0.4min #20-25

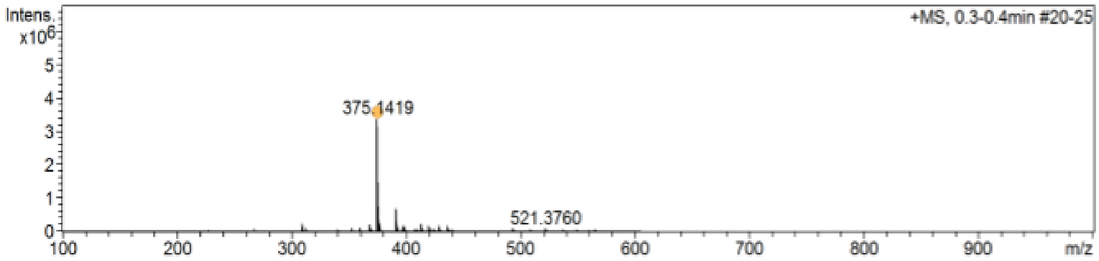

Meas. m/z	#	Ion Formula	m/z	err [ppm]	mSigma	# Sigma	Score	rdb	e⁻ Conf	N-Rule
375.1419	1	C21H19N4O3	375.1452	8.7	4.6	1	21.76	14.5	even	ok
	2	C20H23O7	375.1438	5.1	11.3	2	63.08	9.5	even	ok
	3	C17H15N10O	375.1425	1.6	15.7	3	100.00	15.5	even	ok
	4	C27H19O2	375.1380	-10.5	25.9	4	6.76	18.5	even	ok
	5	C16H19N6O5	375.1411	2.0	26.8	5	71.90	10.5	even	ok
	1	C19H20N4NaO3	375.1428	-2.3	13.7	1	73.41	11.5	even	ok
	2	C18H24NaO7	375.1414	-1.3	22.2	2	100.00	6.5	even	ok

Figure S45. ^{1}H NMR spectrum (500 MHz, CD_3OD) of (**6**)

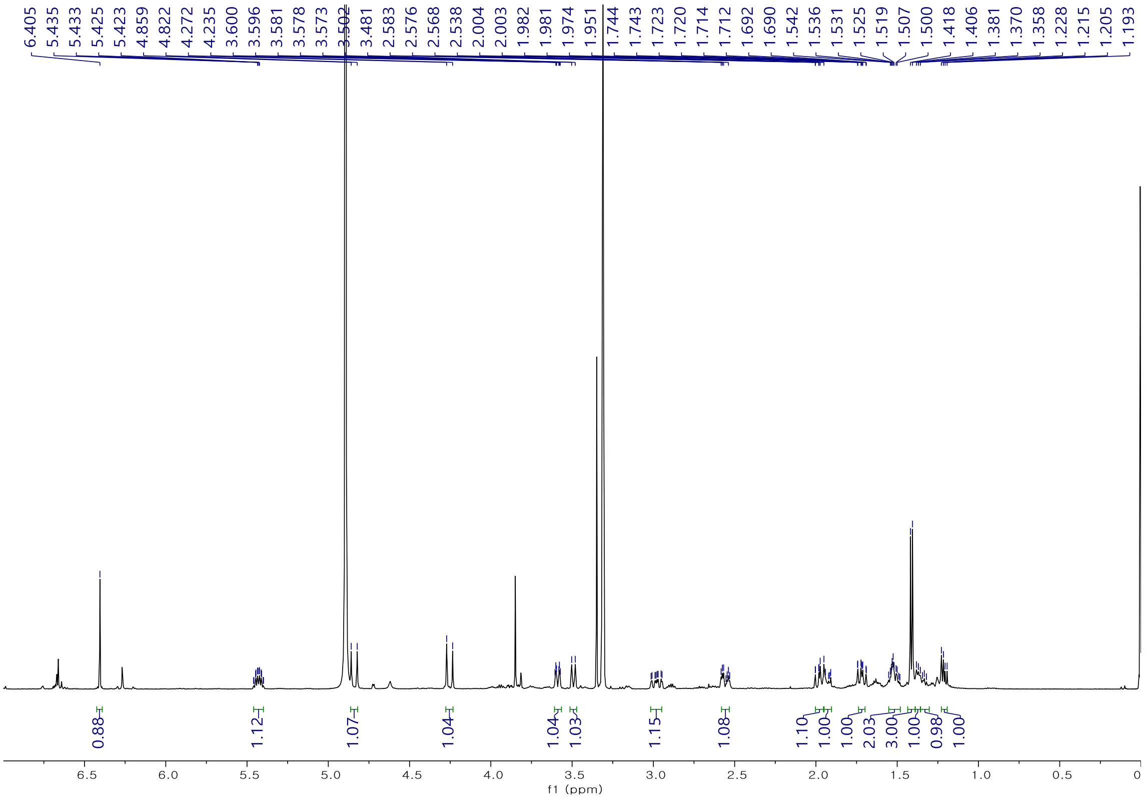

Figure S46. ^{13}C NMR spectrum (125 MHz, CD_3OD) of (**6**)

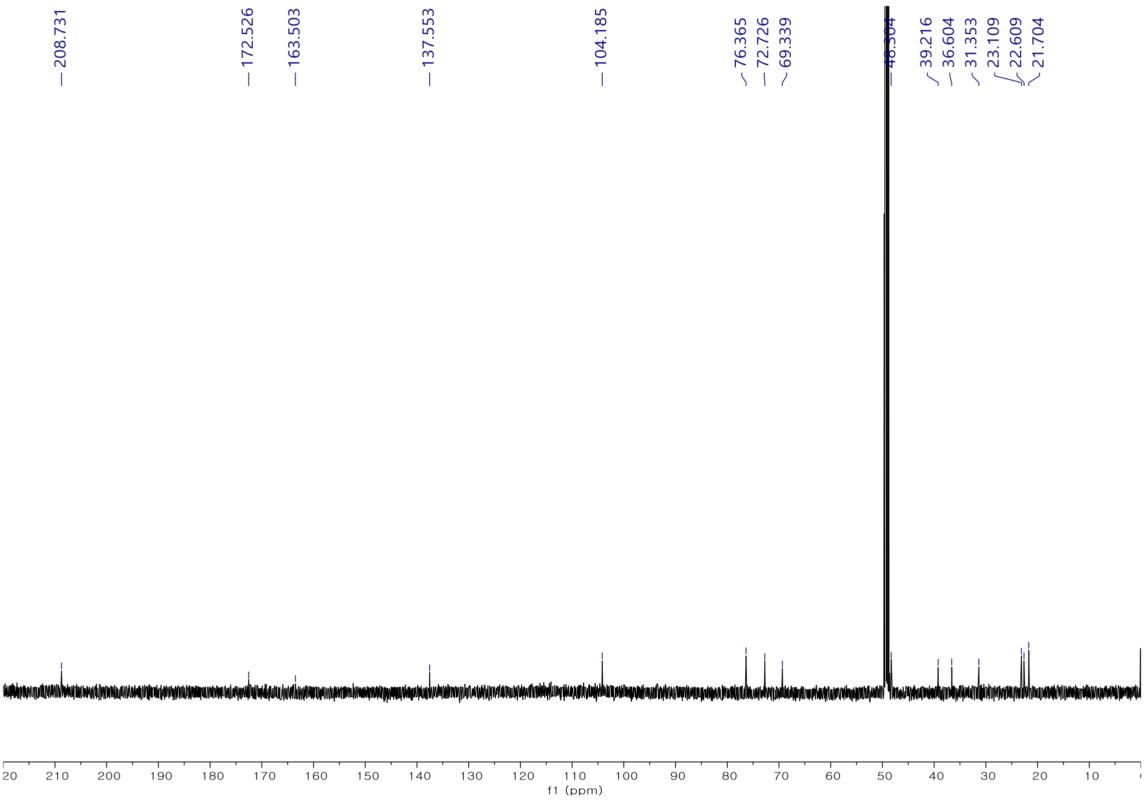

Figure S47. ^1H-^1H COSY spectrum (500 MHz, CD_3OD) of (**6**)

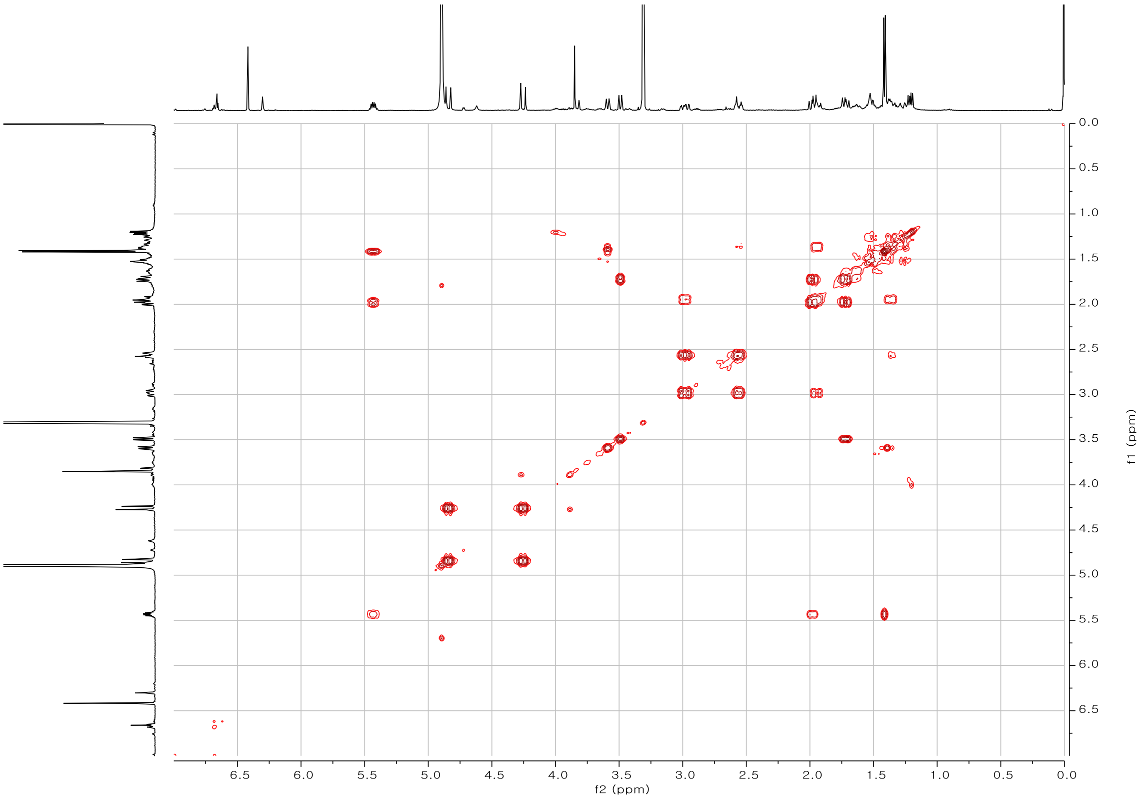

Figure S48. HSQC spectrum (500 MHz, CD_3OD) of (**6**)

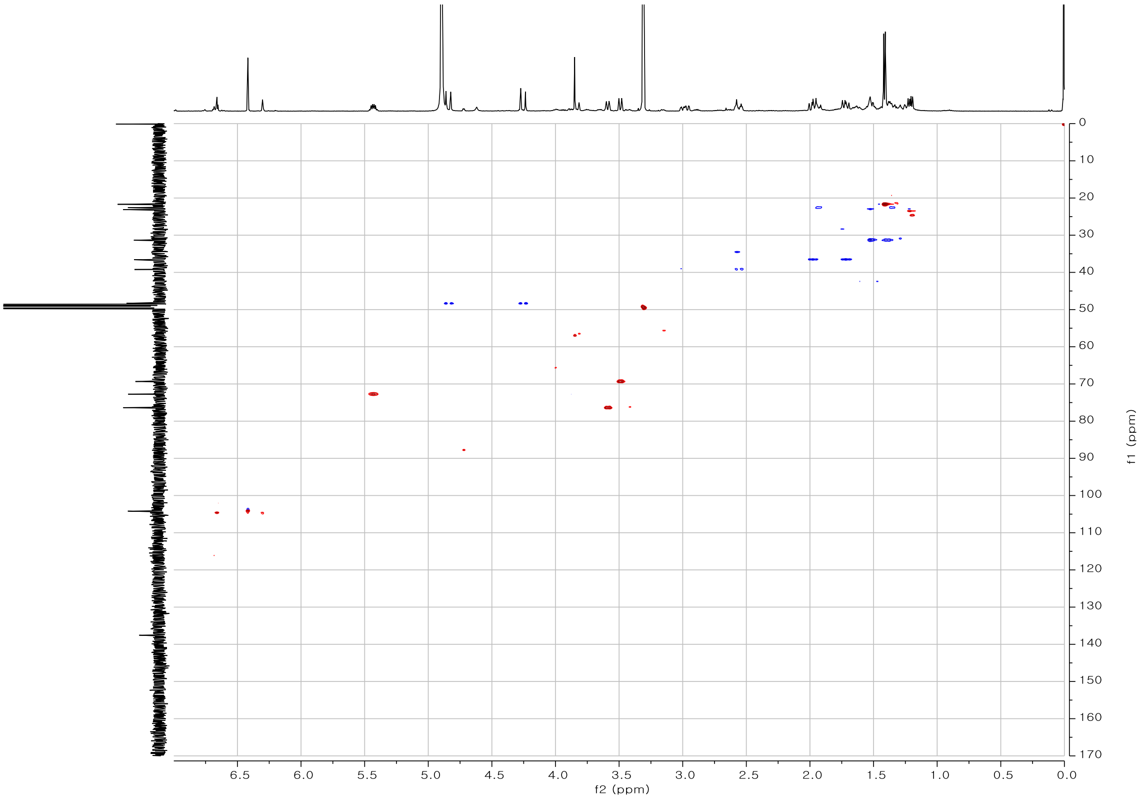

Figure S49. HMBC spectrum (500 MHz, CD_3OD) of (**6**)

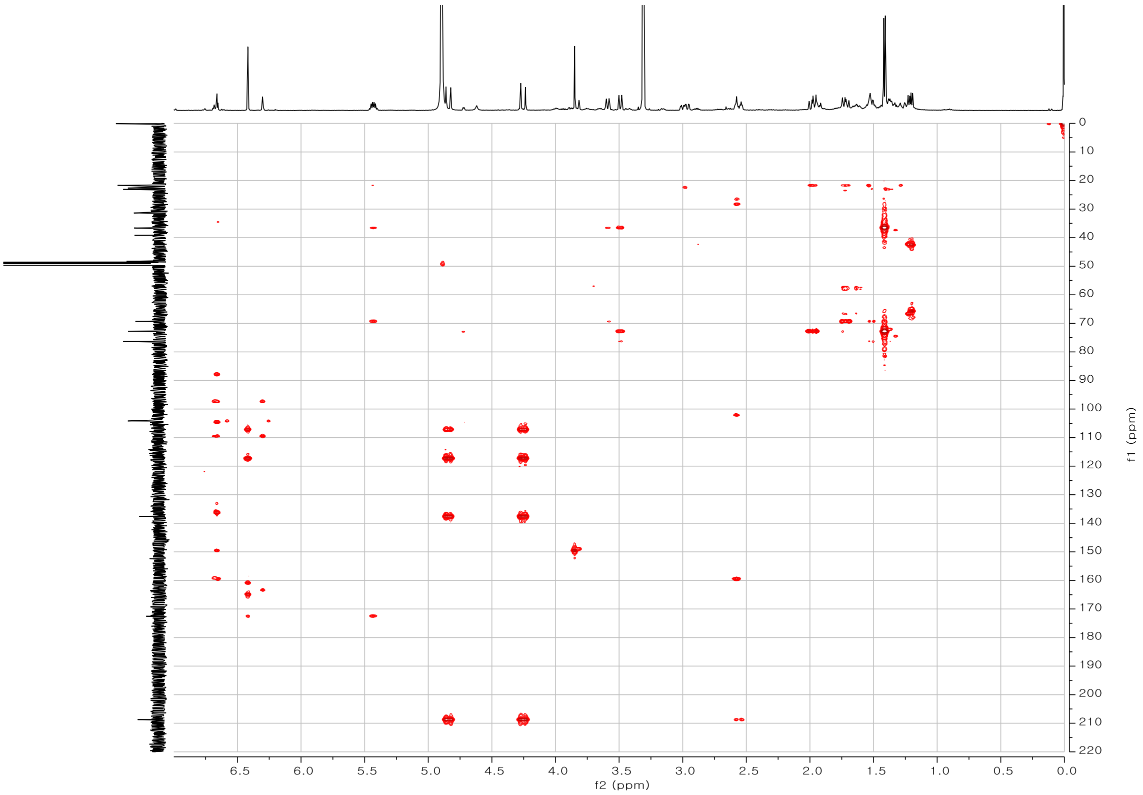

Figure S50. HRESIMS spectrum of (**6**)

Compound Spectrum SmartFormula Report

Analysis Info

Analysis Name	D:\Data\Data\2019\DSWU\SSH\BSH06\BSH002-1.d	Acquisition Date	6/27/2019 5:11:53 PM
Method	Tune_pos_Standard_100_600_190102.d.m	Operator	lee
Sample Name	BSH02_9F3A7	Instrument	compact 8255754.10024
Comment			

Acquisition Parameter

Source Type	ESI	Ion Polarity	Positive	Set Nebulizer	0.4 Bar
Focus	Active	Set Capillary	4000 V	Set Dry Heater	200 °C
Scan Begin	100 m/z	Set End Plate Offset	-500 V	Set Dry Gas	4.0 l/min
Scan End	600 m/z	Set Charging Voltage	2000 V	Set Divert Valve	Source
		Set Corona	0 nA	Set APCI Heater	0 °C

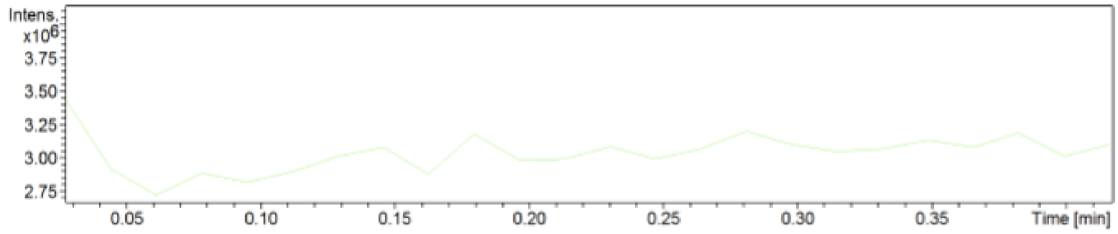

#	RT [min]	Area	Int. Type	I	S/N	Chromatogram	Max. m/z	FWHM [min]
n.a.	0.2	n.a.	Average spectrum	n.a.	n.a.	n.a.	409.1041	n.a.

+MS, 0.1-0.2min #4-14

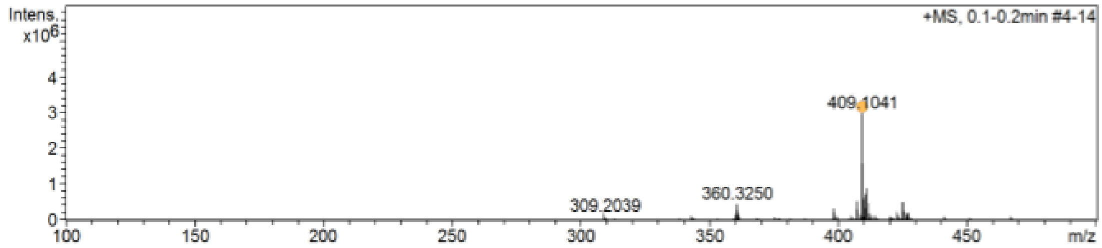

Meas. m/z	#	Ion Formula	m/z	err [ppm]	mSigma	# Sigma	Score	rdb	e⁻ Conf	N-Rule
409.1041	1	C16H18ClN6O5	409.1022	-4.6	21.6	1	55.56	10.5	even	ok
	2	C20H22ClO7	409.1049	1.9	26.4	2	100.00	9.5	even	ok
	3	C15H22ClN2O9	409.1008	7.9	27.1	3	15.18	5.5	even	ok
	4	C21H18ClN4O3	409.1062	5.2	30.2	4	37.81	14.5	even	ok
	5	C15H23Cl2N4O5	409.1040	0.1	157.1	5	0.48	5.5	even	ok
	6	C14H27Cl2O9	409.1027	-3.4	159.2	6	0.20	0.5	even	ok
	7	C20H23Cl2N2O3	409.1080	-9.7	161.5	7	0.02	9.5	even	ok
	8	C14H28Cl3N2O5	409.1058	4.3	269.2	8	0.00	0.5	even	ok
	1	C19H19ClN4NaO3	409.1038	0.7	23.3	1	100.00	11.5	even	ok
	2	C18H23ClNaO7	409.1025	3.9	23.4	2	47.04	6.5	even	ok
	3	C13H24Cl2N4NaO5	409.1016	6.0	155.5	3	0.09	2.5	even	ok
	4	C18H24Cl2N2NaO3	409.1056	3.8	158.0	4	0.14	6.5	even	ok
	5	C17H29Cl3NaO3	409.1074	-8.3	270.2	5	0.00	1.5	even	ok

Figure S51. ^{1}H NMR spectrum (500 MHz, CD_3OD) of (**8**)

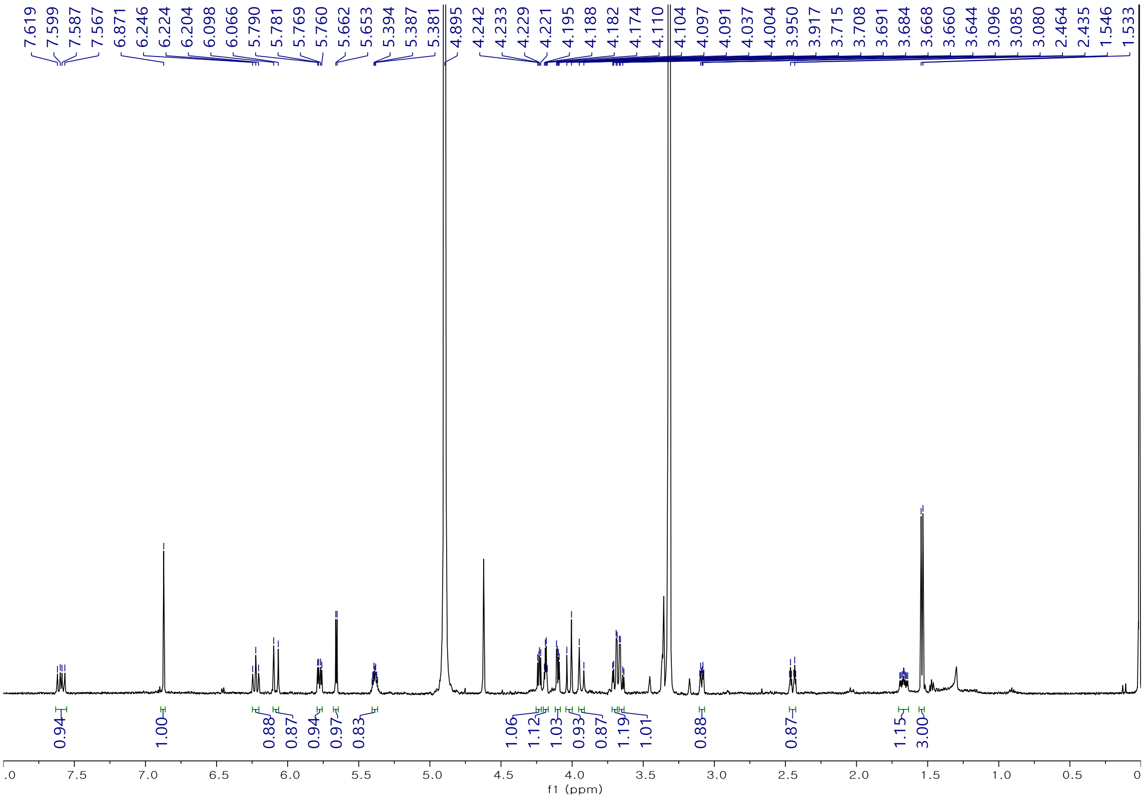

Figure S52. ^{13}C NMR spectrum (125 MHz, CD_3OD) of (**8**)

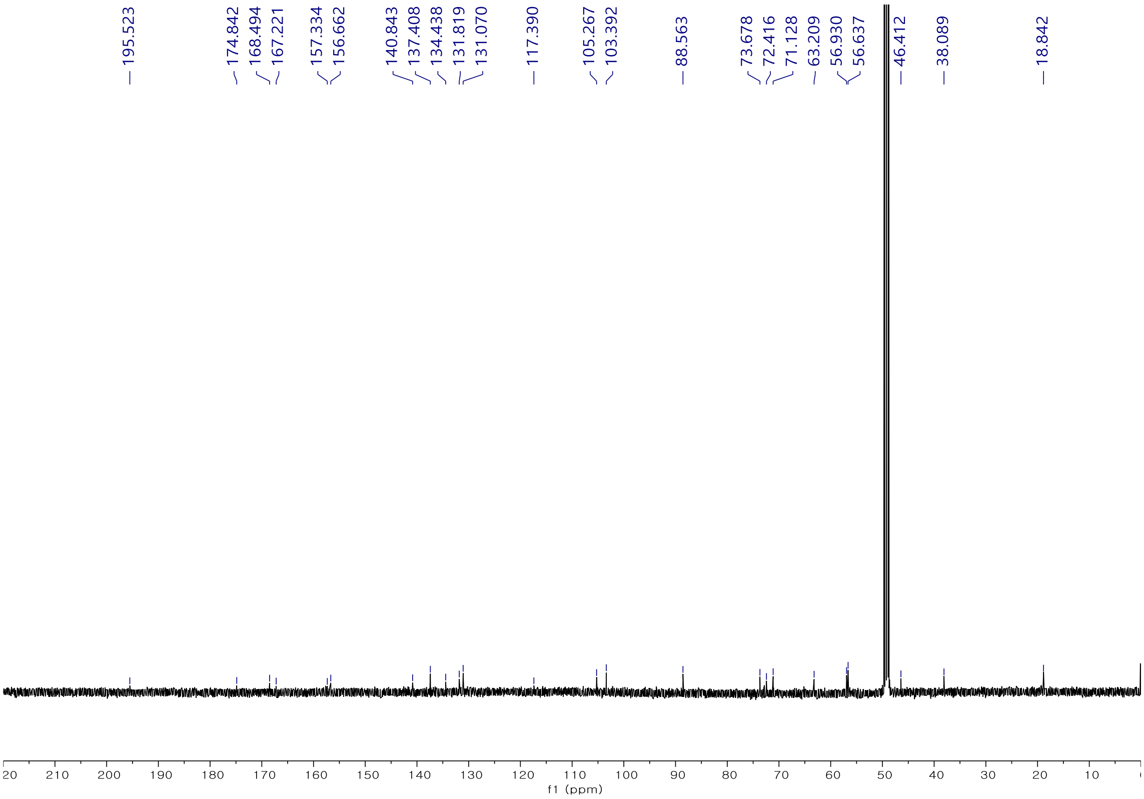

Figure S53. ^{1}H-^{1}H COSY spectrum (500 MHz, CD_3OD) of (**8**)

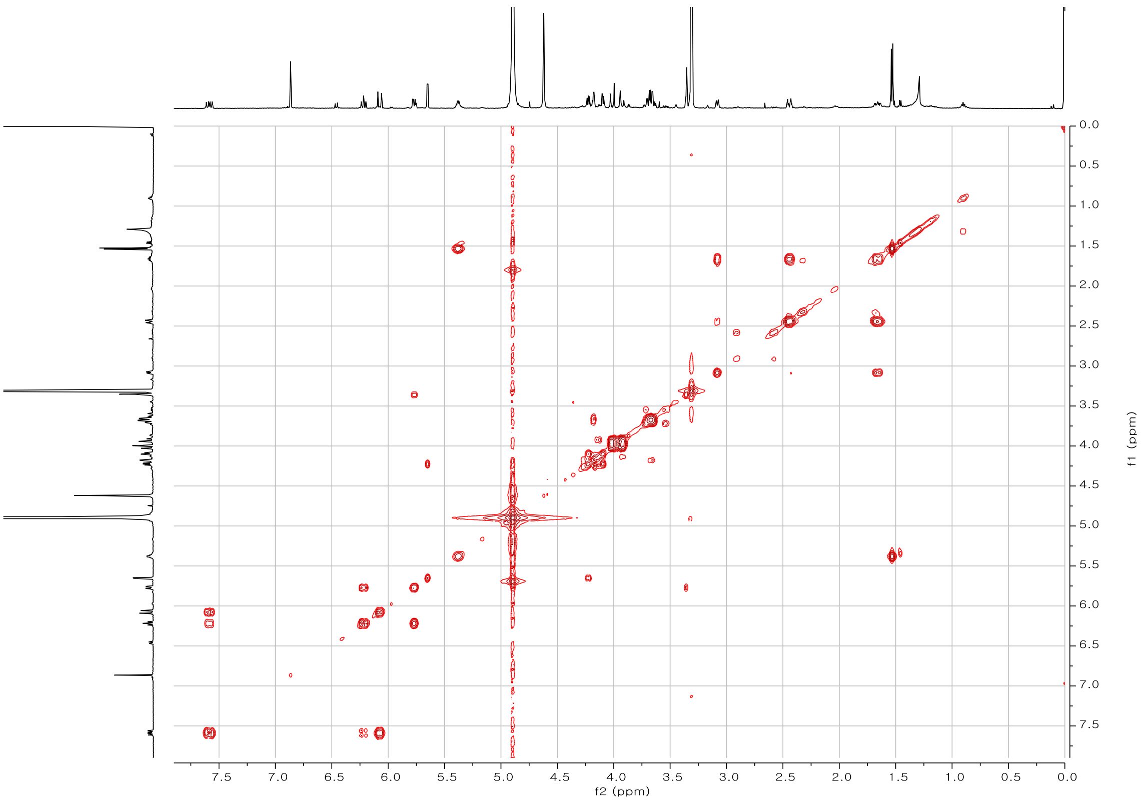

Figure S54. HSQC spectrum (500 MHz, CD_3OD) of (**8**)

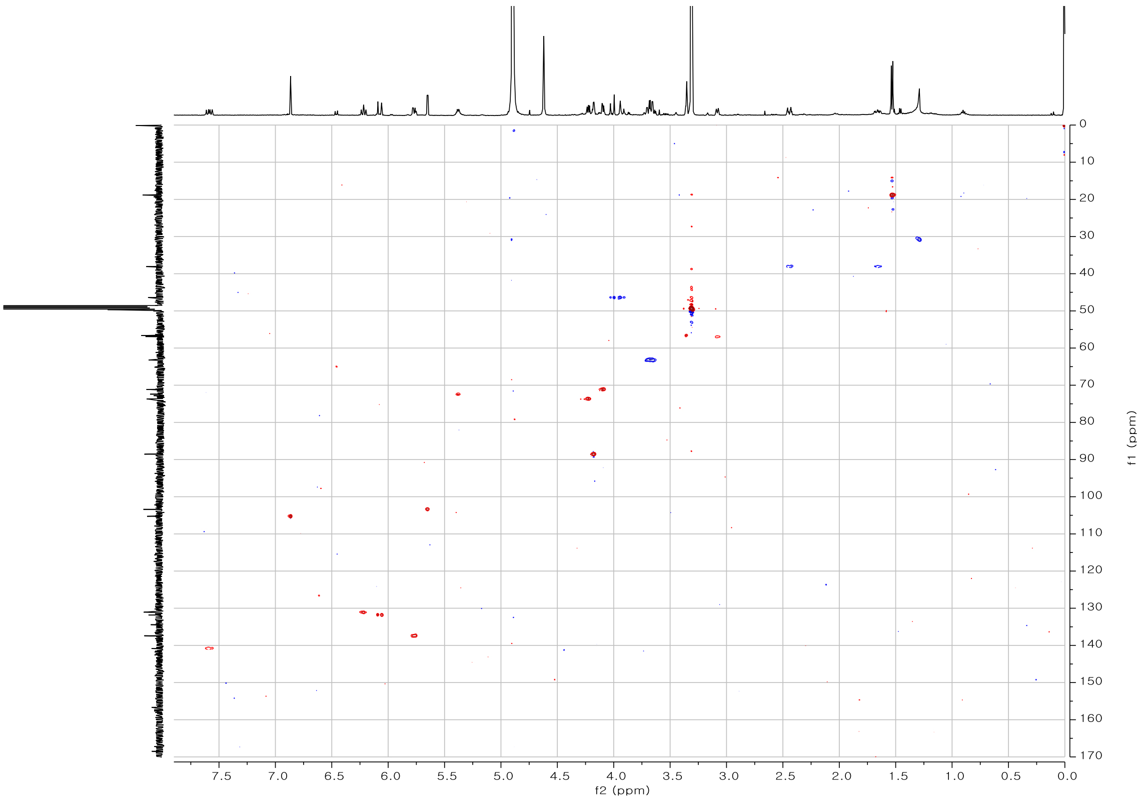

Figure S55. HMBC spectrum (500 MHz, CD_3OD) of (**8**)

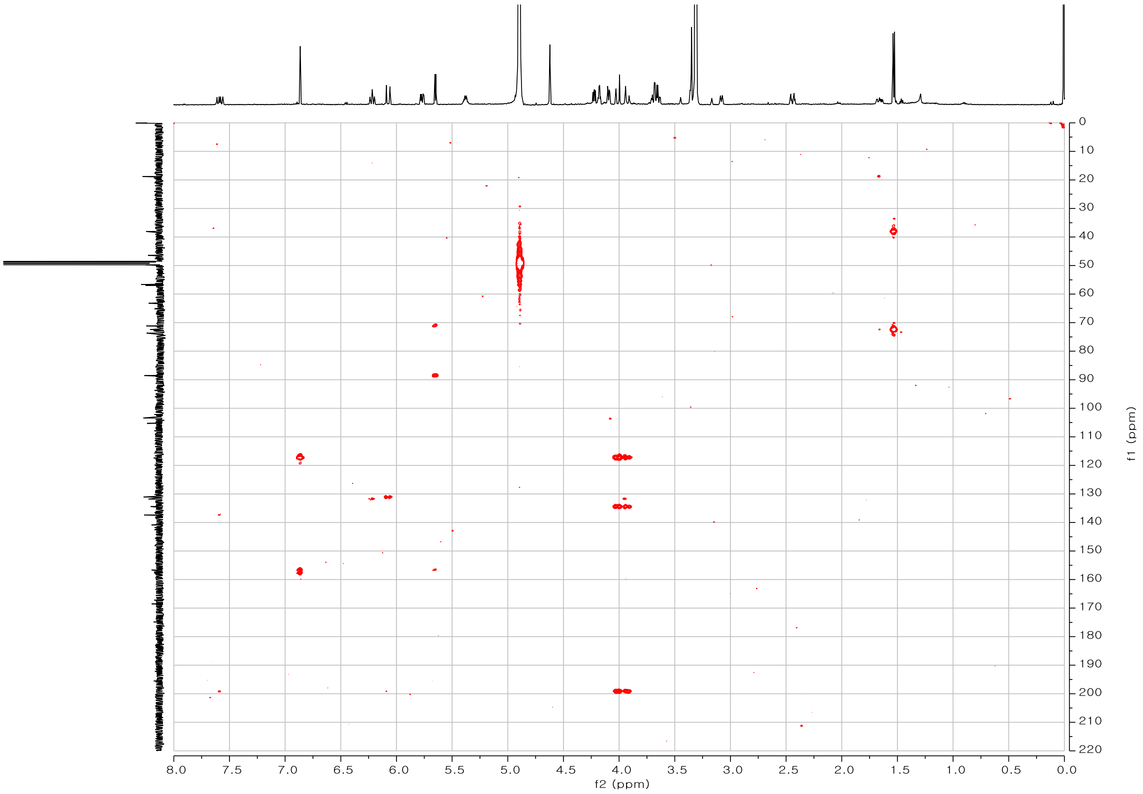

Figure S56. HRESIMS spectrum of (**8**)

Compound Spectrum SmartFormula Report

Analysis Info

Analysis Name	D:\Data\Data\2019\DSWU\SSH\BSH06\BSH004.d	Acquisition Date	6/27/2019 11:56:02 AM
Method	Tune_pos_Standard_100_1000_190102.d.m	Operator	lee
Sample Name	BSH04_9G3E5	Instrument	compact 8255754.10024
Comment			

Acquisition Parameter

Source Type	ESI	Ion Polarity	Positive	Set Nebulizer	0.4 Bar
Focus	Active	Set Capillary	4000 V	Set Dry Heater	200 °C
Scan Begin	100 m/z	Set End Plate Offset	-500 V	Set Dry Gas	4.0 l/min
Scan End	1000 m/z	Set Charging Voltage	2000 V	Set Divert Valve	Source
		Set Corona	0 nA	Set APCI Heater	0 °C

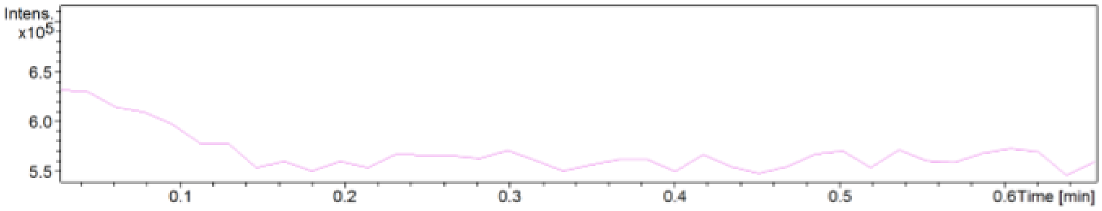

#	RT [min]	Area	Int. Type	I	S/N	Chromatogram	Max. m/z	FWHM [min]
n.a.	0.4	n.a.	Average spectrum	n.a.	n.a.	n.a.	519.1033	n.a.

+MS, 0.3-0.6min #16-35

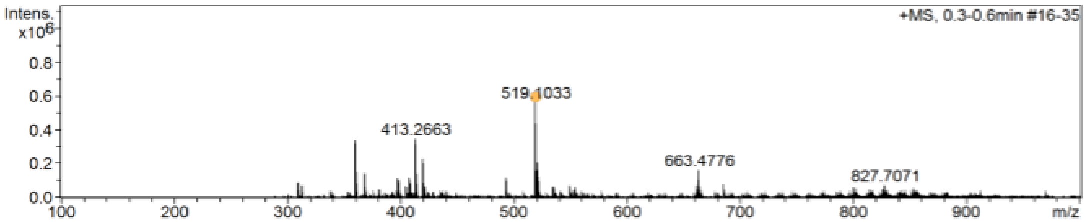

Meas. m/z	#	Ion Formula	m/z	err [ppm]	mSigma	# Sigma	Score	rdb	e⁻ Conf	N-Rule
519.1033	1	C26H19N2O10	519.1034	-0.2	163.1	1	100.00	18.5	even	ok
	2	C27H15N6O6	519.1048	-2.7	165.7	2	41.16	23.5	even	ok
	3	C21H19N4O12	519.0994	7.6	165.9	3	4.11	14.5	even	ok
	4	C22H15N8O8	519.1007	-5.0	167.4	4	14.69	19.5	even	ok
	5	C38H15O3	519.1016	3.4	168.7	5	26.05	31.5	even	ok
	6	C28H11N10O2	519.1061	5.3	168.8	6	11.56	28.5	even	ok
	7	C23H11N12O4	519.1021	2.4	169.4	7	34.65	24.5	even	ok
	8	C24H7N16	519.1034	0.1	171.9	8	53.14	29.5	even	ok
	9	C19H7N18O2	519.0994	7.6	173.6	9	2.32	25.5	even	ok
	1	C29H20NaO8	519.1050	-3.3	163.6	1	72.00	19.5	even	ok
	2	C24H20N2NaO10	519.1010	-4.5	165.2	2	39.68	15.5	even	ok
	3	C30H16N4NaO4	519.1064	-5.8	166.8	3	18.73	24.5	even	ok
	4	C25H16N6NaO6	519.1024	-1.9	167.2	4	86.96	20.5	even	ok
	5	C26H12N10NaO2	519.1037	0.7	169.8	5	100.00	25.5	even	ok
	6	C17H24N2NaO15	519.1069	-6.8	170.3	6	8.52	6.5	even	ok
	7	C21H12N12NaO4	519.0997	7.1	171.4	7	6.83	21.5	even	ok
	8	C22H8N16Na	519.1010	-4.5	173.4	8	21.49	26.5	even	ok
